# Enhancing Blood–Brain Barrier Penetration Prediction by Machine Learning-Based Integration of Novel and Existing, In Silico and Experimental Molecular Parameters from a Standardized Database

**DOI:** 10.1021/acs.jcim.4c02212

**Published:** 2025-03-04

**Authors:** Clemens P. Spielvogel, Natalie Schindler, Christian Schröder, Sarah Luise Stellnberger, Wolfgang Wadsak, Markus Mitterhauser, Laszlo Papp, Marcus Hacker, Verena Pichler, Chrysoula Vraka

**Affiliations:** † Division of Nuclear Medicine, Department of Biomedical Imaging and Image-Guided Therapy, Medical University of Vienna, Vienna, 1090 Austria; ‡ Christian Doppler Laboratory for Applied Metabolomics, Vienna, 1090 Austria; § Department of Computational Biological Chemistry, University of Vienna, Vienna, 1090 Austria; ∥ Department of Pharmaceutical Sciences, Division of Pharmaceutical Chemistry, University of Vienna, Vienna, 1090 Austria; ⊥ Division of Pharmaceutical Chemistry, Department of Pharmaceutical Sciences, Faculty of Life Sciences, University of Vienna, Josef-Holaubek-Platz 2, Vienna, 1090 Austria; # Vienna Doctoral School of Pharmaceutical, Nutritional and Sport Sciences, University of Vienna, Josef-Holaubek-Platz 2, Vienna, 1090 Austria; ¶ MINUTE medical GmbH, Vienna, 1090 Austria; ∇ Joint Applied Medicinal Radiochemistry Facility of the University of Vienna and the Medical University of Vienna, 1090 Vienna, Austria; ○ Center for Medical Physics and Biomedical Engineering, Medical University of Vienna, 1090 Vienna, Austria; ⧫ Institute of Inorganic Chemistry, Faculty of Chemistry, Währinger Strasse 42, 1090 Vienna, Austria

## Abstract

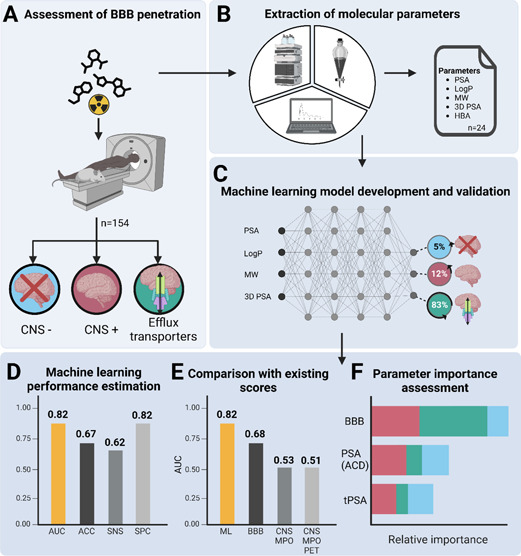

Predicting blood–brain barrier (BBB) penetration is crucial for developing central nervous system (CNS) drugs, representing a significant hurdle in successful clinical phase I studies. One of the most valuable properties for this prediction is the polar surface area (PSA). However, molecular structures are missing geometric optimization, which, together with lack of standardization, leads to variations in calculation. Additionally, prediction rules have been established by combining different molecular properties such as the BBB score or CNS multiparameter optimization (CNS MPO). This study aims to create an approach for 3D PSA calculation, to directly apply this value in combination with a set of 23 other parameters in a novel machine learning (ML)-based scoring, and to further evaluate existing prediction models using a standardized database. We developed and analyzed a standardized data set derived from the same laboratory, encompassing 24 calculated and experimentally determined molecular parameters such as PSA from various models, HPLC log *P* values, and hydrogen bond characteristics for 154 radiolabeled molecules and licensed or well-characterized drugs. These molecules were classified into categories based on BBB penetration, nonpenetration, and interactions with efflux transporters. We supplemented these with a novel in silico 3D calculation of nonclassical PSA. Additionally, we have calculated published prediction rules based on this standardized and transparent database. Using these data, we trained various ML models within a 100-fold Monte Carlo cross-validation framework to derive a novel ML-based prediction score for BBB penetration and validated the three most used existing predictive rules. To interpret the influence of individual molecular parameters and different existing predictive rules, we employed explainable artificial intelligence methods including Shapley additive explanations (SHAP) and surrogate modeling. The ML approach outperformed existing scores for BBB penetration by applying a complex nonlinear integration of molecular properties, with the random forest model achieving the best performance for predicting binary BBB penetration (area under the receiver operating characteristic curve (AUC) 0.88, 95% confidence intervals: 0.87–0.90), and multiclass efflux transporter versus CNS-positive and CNS-negative prediction (AUC 0.82, 95% CI: 0.81–0.82). SHAP analysis revealed the multifactorial nature of the problem, highlighting the advantage of multivariate models over single predictive parameters. The ML model’s superior predictive capability was demonstrated in comparison with existing scoring systems, like the CNS MPO (AUC 0.53), the CNS MPO Positron emission tomography (PET) (AUC 0.51), and BBB score (AUC 0.68) while also enabling the identification of efflux transporter substrates and inhibitors. Our integrated ML approach, combining experimental and in silico measurements with novel in silico methods based on a standardized database including a plethora of different substance groups (licensed drugs and in vivo evaluated PET tracers), enhances the prediction of BBB penetration. This approach may reduce the reliance on extensive experimental measurements and animal testing, accelerating CNS drug development.

## Introduction

The prediction of blood–brain barrier (BBB) penetration is a crucial challenge in the development of central nervous system (CNS) drugs and CNS radiotracers. The BBB is a selective barrier, regulating the passage of compounds from the bloodstream to the brain, making its permeability a critical factor in the efficacy of neuro-pharmaceuticals.

In the history of BBB permeability prediction, significant advances have been made since the introduction of physicochemical parameters and other molecular properties (further abbreviated with molecular/molecule parameters) such as lipophilicity. A crucial aspect has been the exploration of molecular surface properties, particularly the polar surface area (PSA).

Initiatives by van de Waterbeemd and Kansy,^1^ and later by Palm et al.,^2^ highlighted the potential of predicting BBB penetration through computational methods, focusing on the dynamic interactions of molecular surfaces. These methodologies, while insightful, faced limitations in accurately capturing the complex nature of molecular interactions. Later approaches by Clark and Ertl et al. offered more rapid and practical computational methods, yet with limitations regarding molecular conformations and interactions. Stenberg et al.’s partitioned total surface area method^3^ further refined this predictive process by focusing on molecular surface properties most correlated with intestinal drug permeability, indicating a continuous evolution toward more efficient and accurate prediction models for drug permeability, particularly for CNS applications. Despite later and computationally more advanced BBB penetration predictions,^4−6^ challenges persist in efficiently selecting viable drug candidates in the costly and lengthy CNS drug development process. While the topological PSA (tPSA) was used as a single parameter or in combination with the log *D* (pH7.4) value to predict passive diffusion through the BBB, a plethora of rules were published since the 1990s with limitations in data amount, standardized methods (calculations and software), only in silico parameters, no ground truth with regard to degree of BBB penetration, compounds from same substance class or molecular weight (MW) class (<500 Da), and exclusion or lack of description of compounds showing interactions with efflux transporters.^7−11^ The use of multiparameter optimization (MPO) scores, comprising multiple molecular, and physicochemical parameters in one prediction model, such as CNS multiparameter optimization (CNS MPO score),^11^ CNS MPO Positron emission tomography (PET) score,^12^ and BBB score,^13^ led to a more holistic approach on BBB penetration prediction, but still lacks standardization.

Recently, machine learning (ML) has emerged as a promising approach for inferring biomolecular behavior from molecular characteristics, including enhancing BBB penetration prediction.^14,15^ ML algorithms can analyze complex data sets, uncover hidden patterns, and make predictions about new compounds with greater accuracy and speed compared to traditional methods. A critical aspect of ML-based prediction is the quality and diversity of the data sets used for training. As a result, ML models for BBB penetration prediction are ideally trained on experimentally generated results, for example, by employing radiolabeled molecules.

PET has the ability to visualize a molecule’s biodistribution and, hence, its ability to penetrate the BBB in vivo. However, the complex and expensive process associated with the development of novel radiotracers does not allow for PET-based high throughput screening approaches to assess the BBB penetration of large databases of molecules directly.

In this study, we (I) exploit a standardized database of thoroughly investigated drugs including experimental and calculated molecular parameters and known BBB penetration. (II) We explore a novel in silico method to calculate a 3D PSA value and (III) use ML to predict the penetration of the BBB based on a molecule’s properties. (IV) We further employ an explainable artificial intelligence approach to determine each individual parameter’s contribution to the prediction. Lastly, we compared the ML approach with the predictive ability of individual parameters alone and existing multiparameter prediction scores^11−13^ for three drug categories: BBB penetrating (CNS positive), BBB nonpenetrating (CNS negative), and interactions with efflux transporter substrates and compared it to previously published prediction scores.

## Methods

### 3D PSA Calculation

For the calculation of the 3D PSA, first, a force field optimization was performed by using Avogadro 1.2.0 for all molecules. A Merck molecular force field was set up and a geometry optimization with 9999 steps and a steepest descent algorithm with a convergence threshold of 10^–7^ was completed three times for each molecule. All geometry optimization calculations were performed by using density functional theory with B3LYP hybrid functionals employing a 6-31 G(d) basis set. For molecules with delocalized π systems, a D3 dispersion correction was applied. Since the 6-31 G(d) basis set only employs atoms from hydrogen to Krypton, molecules containing Iodine were calculated with the LanL2DZ basis set. Calculations were performed using PyMOL2. In all instances, the solvent radius was defined as 1.4 Å, which is the standard for water. To ensure the accuracy of the calculations, the dot density was adjusted to four, and the dot solvent was set to one to generate dots for the solvent-accessible surface. The whole surface area in Å^2^ accessible to solvent was calculated. The polar atoms used for PSA calculations were selected based on their partial charges of either larger than 0.6 or smaller than −0.6. Finally, 3D PSA calculations considered nitrogen or oxygen atoms, including adjacent hydrogen atoms.

### Parameter Collection

CNS MPO score^11^ and BBB score^13^ were calculated using MarvinSketch 23.8 by Chemaxon. CNS MPO PET^12^ score was calculated using equations provided by Zhang et al.

3D PSA, which is newly introduced by the authors of this work (see chapter above), tPSA^16^ (ChemDraw 20.1.1), and PSA (ACD) were studied. The tPSA is derived from a prediction method in which molecular fragments of polar surface contributions are summarized, which were obtained by least-squares fitting to a single low-energy conformer of the 3D PSA.^16^ The 3D PSA was calculated by using Boltzmann-weighted distribution of low energy conformers and including the van der Waals surface of oxygen, nitrogen, sulfur, and phosphorus with their hydrogen atoms. For the tPSA, 43 different polar atom fragments were created, in which oxygen and nitrogen, as well as phosphorus and sulfur including adjacent hydrogen atoms, were considered polar. PSA (ACD) comprises values calculated with ACD/Laboratories (Royal Society of Chemistry 2024) predictions via the Chemspider Web site, which differ from tPSA values. ACD/Laboratories does not further elucidate prediction parameters or computation details. The log *P* and Clog *P* values were collected from ChemDraw 20.1.1. Other molecular parameters for subsequent ML modeling were collected using ACD (ChemSpider 2023 or ChemSketch 2020.1.2) for MW, freely rotatable bonds, hydrogen bond donor (HBD), hydrogen bond acceptor (HBA), log *P*, log *D* (pH 7.4), HPLC log *P*_ow_μ_pH7.4_-HBA, HBA + HBD, log *P*-HBA, and log *D*_pH7.4_-HBA. The experimental values, HPLC log *P*_ow_μ_pH7.4_, membrane coefficient (K_IAM_), permeability (P_m_) percent human serum albumin (HSA) binding (%HSA), and the logarithm of apparent affinity constant (log *K*) were gathered based on previous studies^17,18^ using immobilized artificial membrane and HSA bioaffinity chromatography.

The p*K*_a_ was computationally predicted using Chemicalize software (Chemaxon Ltd., Budapest, Hungary). The macro p*K*_a_ values were determined over a range of 0–16, with a static acid/base prefix. Tautomerization and resonance effects are not taken into account in the software. Predictions were made under standard conditions of 25 °C and zero ionic strength. The list of all 154 molecules, including all 24 parameters and predictive values and the associated information on in vivo permeability of the BBB public via https://osf.io/cvhe9.

### Target Definition for ML Modeling

As the database included mostly radiotracers and well-described or licensed drugs, we established the ground truth for ML based on the presence or absence of brain uptake including blocking studies for efflux transport ligands using BCRP and P-gp inhibitors such as tariquidar.^19^ Published human and brain tracer biodistribution data were considered. If human data were not available, published data from preclinical imaging studies^20,21^ (murine models) were used. Two prediction targets were employed, including a two-class classification discerning only between molecules that did or did not penetrate the BBB as well as a multiclass classification, additionally discerning efflux transporters.

### Machine Learning

To ensure robust estimation of performance, we employed a 100-fold Monte Carlo cross-validation approach. The general process of Monte Carlo cross-validation is explained in Supporting Information Table S2. In each fold, we randomly allocated 80% of the samples to the training set, while the remaining 20% constituted the test set. All modeling procedures were executed on either the complete training data set or a subset thereof. The test data were solely utilized to assess the performance of the trained models. Within each fold, we conducted fold-specific preprocessing, including feature standardization, feature imputation using k-nearest neighbor imputation, feature selection employing the minimum redundancy maximum relevance (mRMR) algorithm,^22^ addressing imbalances through the synthetic minority oversampling technique,^23^ and automated hyperparameter optimization via random search. The ML models were then trained on the preprocessed training data. We employed a total of six classification algorithms, including support vector machine, logistic regression, k-nearest neighbors, random forest, extreme gradient boosting, and explainable boosting machine.^24^ Performance metrics for evaluation included the area under the receiver operating characteristic (AUC), accuracy (ACC), sensitivity (SNS), specificity (SPC), positive predictive value, and negative predictive value. For the multiclass classification, a one-versus-rest approach was used to calculate metrics. The multiclass AUC was calculated using macro averaging. We calculated 95% confidence intervals (CI) for all of the cross-validation results. Metric formulas are provided in the Supporting Information, Metrics. For ML, Python 3.9.5 (Python Software Foundation) was used along with the packages NumPy,^25^ Pandas,^26^ Scikit-learn,^27^ Imbalanced-learn,^28^ Shap,^29^ XGBoost,^30^ InterpretML,^24^ UMAP,^31^ and mRMR.^22^

### Interpretability Methods

In addition to inherently interpretable models such as decision trees, logistic regression, and explainable boosting machine classifiers, we incorporated various explainable artificial intelligence (XAI) techniques. Shapley additive explanations (SHAP) is a method in ML that allows one to explain the output of a model on a feature-by-feature basis while still considering feature interactions.^32^ SHAP scores the importance of each feature by attributing the prediction of each sample to individual features based on cooperative game theory. SHAP was applied to elucidate feature importance and investigate the directionality and extent of impact on outcomes.^29^ SHAP features were calculated on a “final” model, which was trained on the entire data set after performance estimation using Monte Carlo cross-validation. To enhance the interpretability of opaque models such as random forest and neural networks, we constructed surrogate decision tree models. These surrogate models were trained to predict the output of the opaque model, providing transparency in the prediction process while preserving predictive performance.

### Comparative Assessment of Existing Scores

The developed predictive performance of the best-performing ML model was compared with the established CNS MPO, CNS MPO PET, and BBB score. The prediction of efflux transporter interactions (substrates and inhibitors) has been considered understudied. They build a third and very heterogeneous category of drugs. Additionally, classification in substrates and inhibitors is for some compounds difficult as this can depend on the concentration. However, substrates can be defined as passive permeable but underly an active transport back to plasma compartment. Hence, we performed two comparisons. First, a comparison of the developed ML model, CNS MPO, CNS MPO PET, and BBB scores for all molecules included in this study. Second, a comparison of the ML model, CNS MPO, CNS MPO PET, and BBB score only for CNS positive versus CNS negative substances, where efflux transporter substrates were categorized as CNS positive. For the BBB score, we employed a cutoff value of 4 as was previously determined to be associated with CNS positivity.^13^ For CNS MPO and CNS MPO PET scores, previously proposed thresholds of 4 and 3 were employed, respectively.^7,11^

### Statistical Analysis

Continuous data are expressed as the mean with standard deviation or as median with interquartile range (IQR); categorical variables are presented as numbers and percentages. 95% CIs of the ML performance were calculated over the 100 Monte Carlo cross-validation folds. *P* values smaller than 0.05 were considered statistically significant. For the comparison of molecular properties between the three groups, CNS negative, CNS positive, and efflux transporter substrate, *p* values were calculated using *t*-test if distributions were normal based on the Shapiro Wilks test; otherwise, a Mann–Whitney *U* test was used for comparison. Statistical analysis was performed using Python 3.9.5 and the packages SciPy,^33^ NumPy,^25^ and Pandas.^26^

## Results

**Figure 1:**
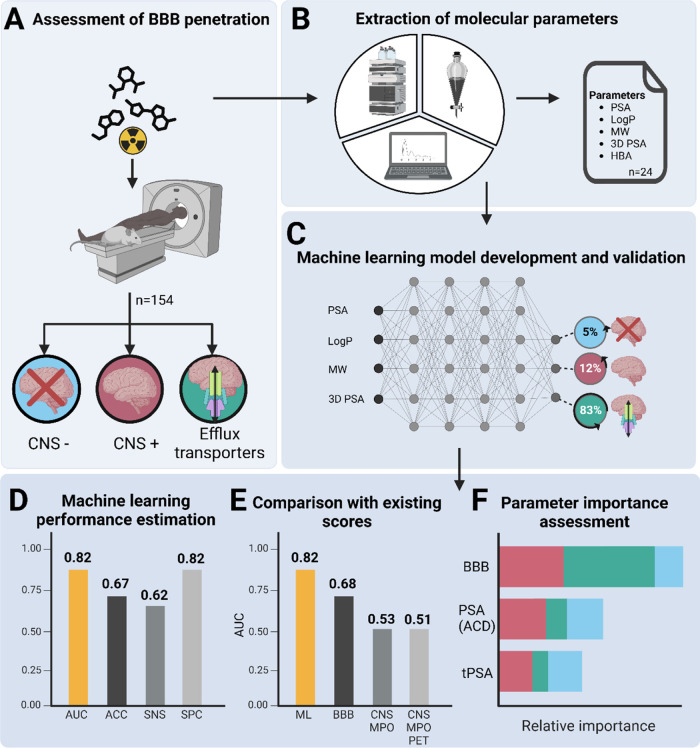
Graphical abstract. (A) Overall, 154 drugs and radioactively labeled molecules were assessed for their ability to penetrate the BBB, including the identification of efflux transporter substrates. (B) In silico and experimental molecular parameters and existing scores were measured and computed for each molecule. (C) ML models were trained and evaluated for the prediction of BBB penetration. (D) ML performance for the multiclass classification was high (AUC 0.82). (E) The ML model outperformed existing scores in the multiclass classification, differentiating CNS positive, CNS negative and efflux transporter substrates. (F) Relative importance of the three most important prediction parameters (BBB score, PSA (ACD), and tPSA) in the ML model. Created using BioRender.

Of the 154 molecules included in the analysis, 42 (27.3%) did not penetrate the BBB, 68 (44.2%) did penetrate the BBB, and 44 (28.6%) were determined to be efflux transporter substrates or inhibitors. All molecules and features stratified by CNS positivity are listed in Supplementary Tables S1 and S2 including references of their use in clinical or preclinical studies or a self-report for the failed radiotracers. A conceptual overview of the study is depicted in Figure 1.

## 3D PSA Calculations

**Figure 2:**
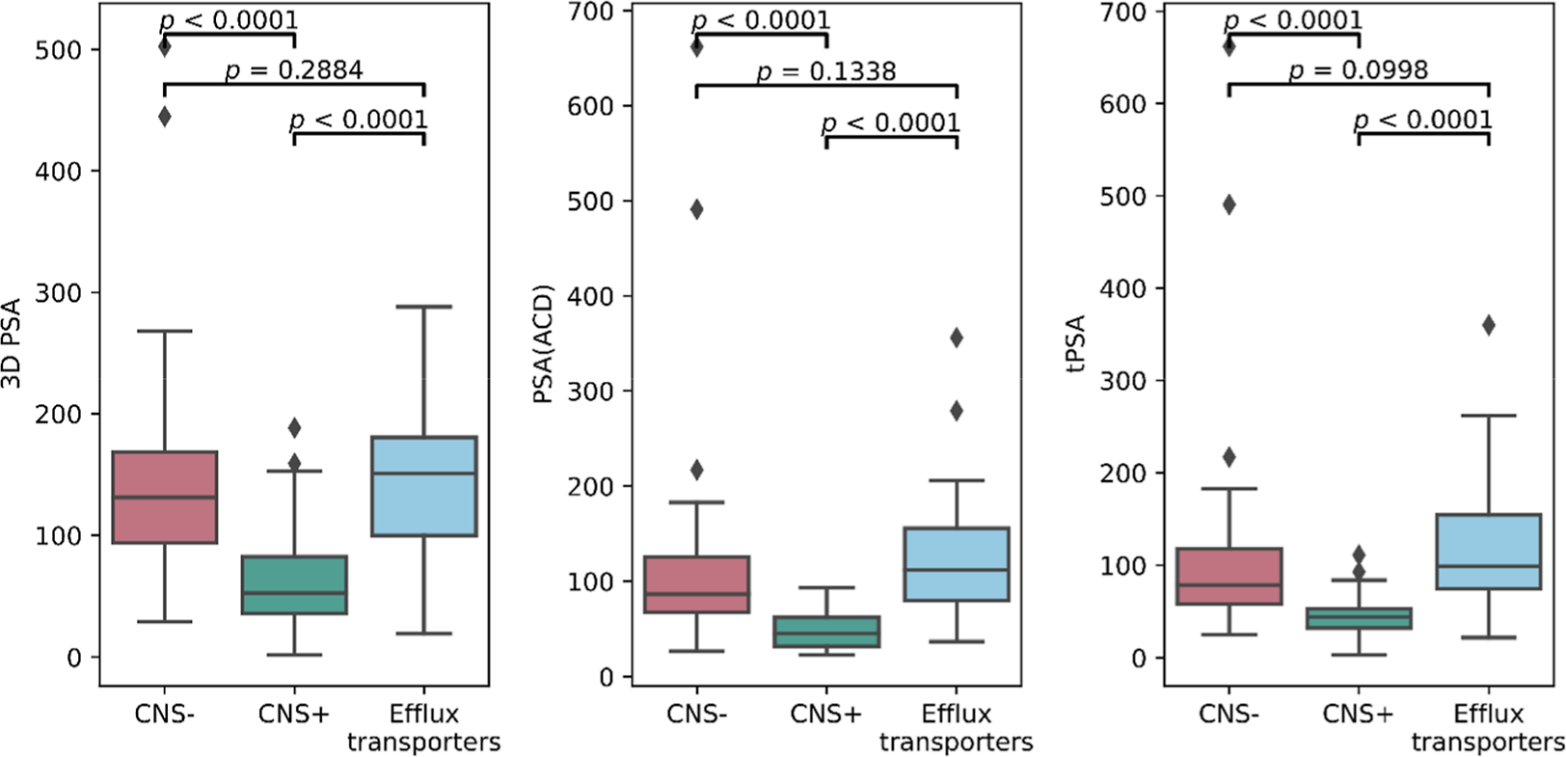
Comparison of distributions of the three calculated PSA values for each class (CNS+, CNS–, and efflux transporter substrates). *P* values were calculated using *t*-test if distributions were normal based on Shapiro Wilks test, otherwise, a Mann–Whitney *U* test was used for comparison.

The 3D PSA was calculated for 154 molecules from quantum-chemically optimized structures. 3D PSA values ranged between 62.9 (±34.7) Å^2^ for BBB penetrating (*n* = 68), 146.2 (±94.4) Å^2^ for BBB nonpenetrating molecules (*n* = 42), and between 149.6 (±68.6) Å^2^ for molecules within the efflux transporter group (*n* = 44). Pairwise Mann–Whitney *U* tests were performed for the comparison between each combination of CNS positive, CNS negative, and efflux transporter groups. There was a significant difference between CNS positive versus negative (*p* value < 0.0001) and CNS positive versus efflux transporter substrates (*p* value < 0.0001); however, there was no significant difference in the comparison between CNS negative and efflux transporter substrates (*p* value 0.2884) (Figure 2).

### ML Performance

**Figure 3:**
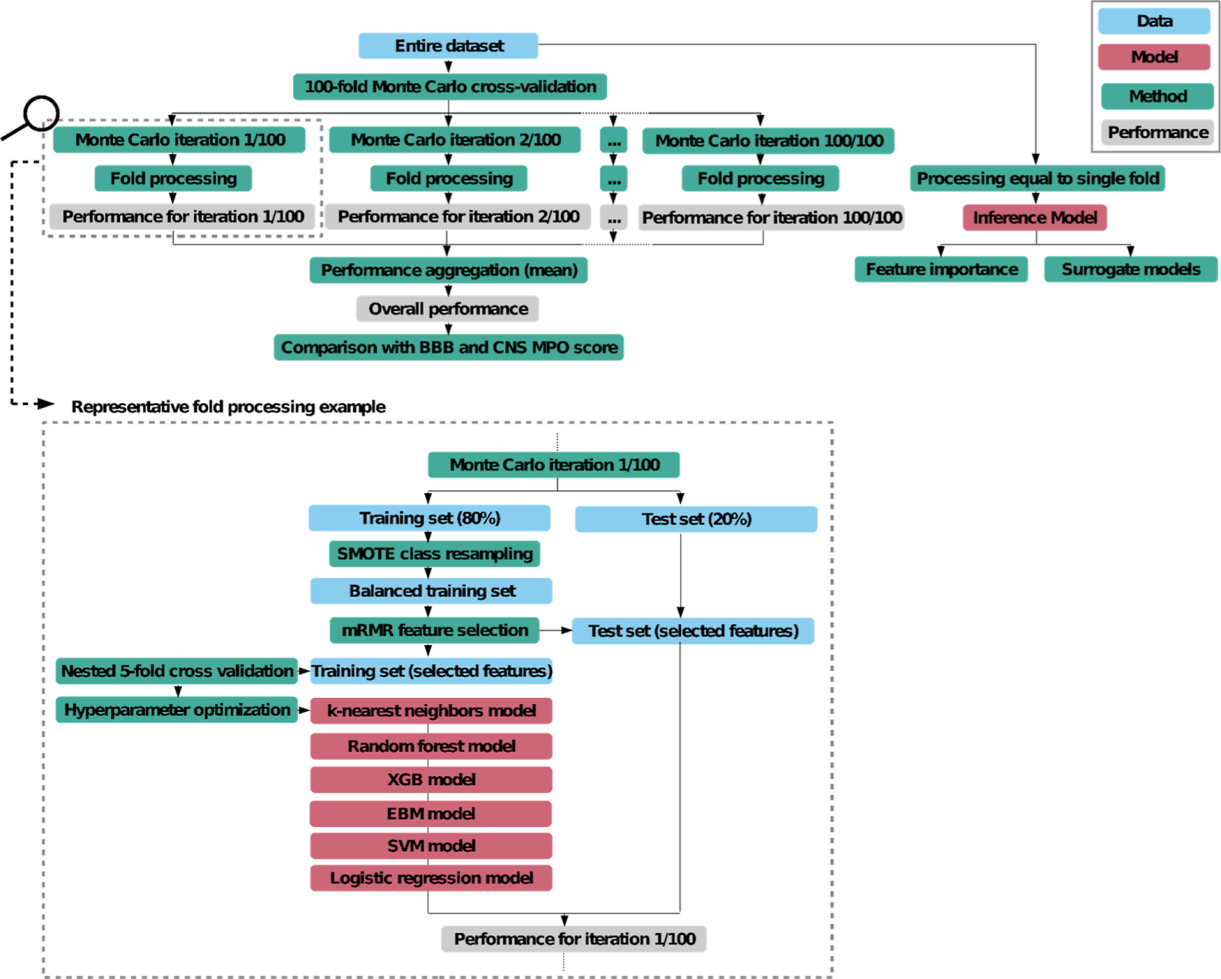
ML workflow. SMOTE = synthetic minority oversampling technique for class balancing. mRMR = minimum redundancy maximum relevance feature selection algorithm. XGB = extreme gradient boosting. ebm = explainable boosting machine. svm = support vector machine.

An overview of the ML workflow is shown in Figure 3. Of the six investigated ML models, the best performance for both prediction tasks was achieved by the random forest model, followed by the explainable boosting machine model (Table 1). The random forest model achieved performances of AUC 0.82 (95% CI 0.81–0.82), accuracy (ACC) 0.67 (95% CI 0.65–0.69), sensitivity (SNS) 0.62 (95% CI 0.60–0.63), and specificity (SPC) 0.81 (95% CI 0.80–0.83), for the multiclass prediction differentiating CNS positive, CNS negative, and efflux transporter substrates. However, despite the strong overall performance of the multiclass classification model, the performance of efflux transporter substrates alone was only AUC 0.57 (95% CI 0.52, 0.61). For the two-class prediction differentiating exclusively between CNS positive and negative compounds, the model achieved even higher performances with AUC of 0.88 (95% CI 0.84–0.90), ACC of 0.83 (95% CI 0.80–0.85), SNS of 0.88 (95% CI 0.84–0.89), and SPC of 0.75 (95% CI 0.71–0.78).

**Table 1:** ML Classification Performance Metrics for the Two-Class and Multi-Class Prediction

	target	ACC	SNS	SPC	PPV	NPV	AUC
k-nearest neighbors	two-class	0.83	0.89	0.72	0.85	0.80	0.84
**random forest**	two-class	**0.83**	**0.88**	**0.75**	**0.87**	**0.79**	**0.88**
extreme gradient boosting	two-class	0.82	0.86	0.74	0.86	0.77	0.86
explainable boosting machine	two-class	0.82	0.88	0.72	0.85	0.79	0.87
support vector machine	two-class	0.83	0.90	0.70	0.84	0.83	0.87
logistic regression	two-class	0.82	0.88	0.73	0.85	0.80	0.86
k-nearest neighbors	multiclass	0.68	0.63	0.82	0.65	0.65	0.80
**random forest**	**multiclass**	**0.67**	**0.62**	**0.82**	**0.63**	**0.63**	**0.82**
extreme gradient boosting	multiclass	0.67	0.62	0.81	0.63	0.63	0.81
explainable boosting machine	multiclass	0.65	0.59	0.81	0.60	0.60	0.81
support vector machine	multiclass	0.64	0.58	0.79	0.63	0.63	0.77
logistic regression	multiclass	0.66	0.61	0.80	0.61	0.61	0.78

### ML Parameter Weighting

**Figure 4:**
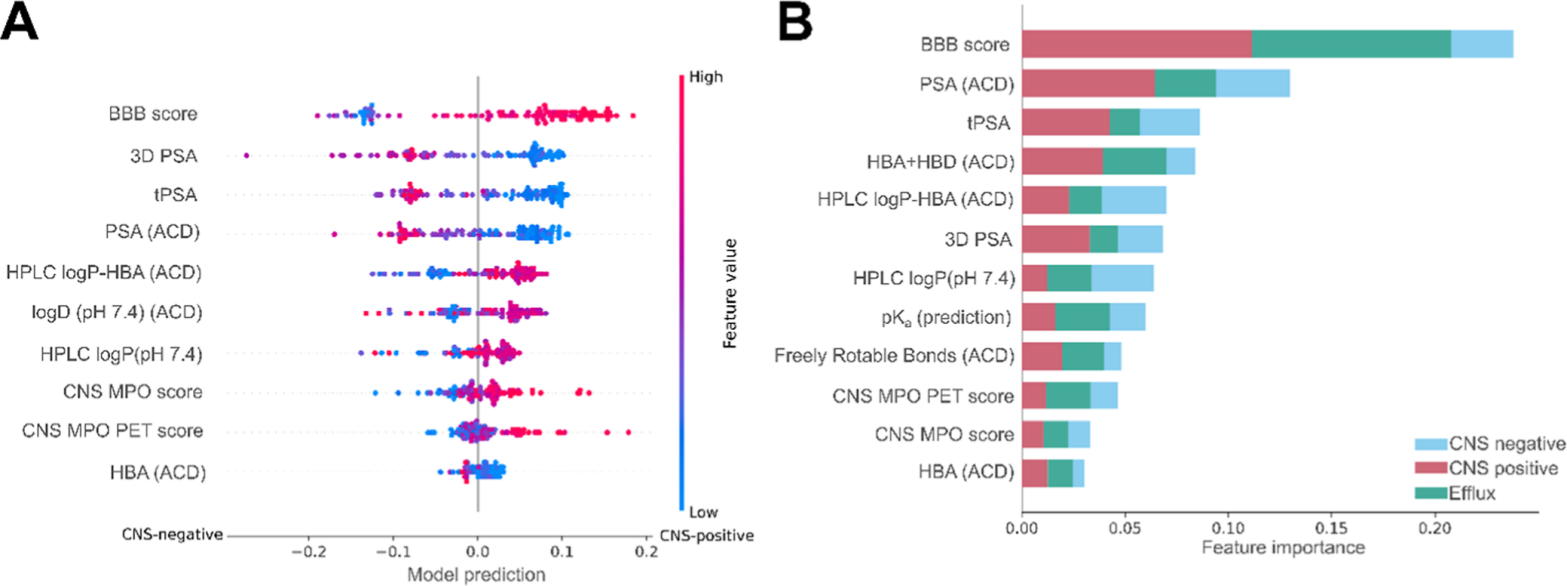
SHAP feature importance for each predicted class in the two-class (A) and multiclass (B) prediction. The subfigures show the parameters selected by each model ordered from the most to the least important from top to bottom. Overall, subfigure A provides a visual guidance indicating whether molecules with higher or lower values for a given feature are more likely to be predicted as CNS-positive or -negative. Each dot represents a molecule. The color of the dot indicates whether a molecule had a comparatively high or low value for the feature in the given row (*y*-axis). Molecules with a model prediction (*x*-axis) less than 0 had a tendency to be predicted as CNS-negative while molecules with a feature importance value larger than 1 were likely to be predicted as CNS-positive. The higher the absolute *x*-axis value, the higher the importance of the given feature for the prediction. In subfigure B, multiclass SHAP importance is shown for each parameter with respect to the importance for each individual class. For example, while HBA + HBD (ACD) had an overall lower importance compared to tPSA, HBA + HBD (ACD) was substantially more important for the identification of efflux transporter substrates.

For the two-class classification differentiating only CNS positive versus negative substances, the BBB score was ranked as the most important feature by the Shapley additive explanations (SHAP) importance assessment followed by the novel 3D PSA and tPSA (derived from ChemDraw). The CNS MPO and CNS MPO PET scores were only ranked as the eighth and ninth most important feature, respectively (Figure 4A).

For the multiclass classification, SHAP analysis revealed the consistent importance of the BBB score, closely followed by the PSA (ACD), tPSA, and HBA as well as HBD (ACD) (Figure 4B). Like in the two-class classification, the 3D PSA ranked as the second most predictive feature; however, it ranked as the sixth most important in the multiclass classification. The BBB score showed substantially higher importance for molecules penetrating the BBB and efflux transporters compared with all other features. On the other hand, the PSA (ACD) had the highest predictive capabilities for the identification of molecules that do not penetrate the BBB; however, it was closely followed by the BBB score and the tPSA.

### Surrogate Models

**Figure 5:**
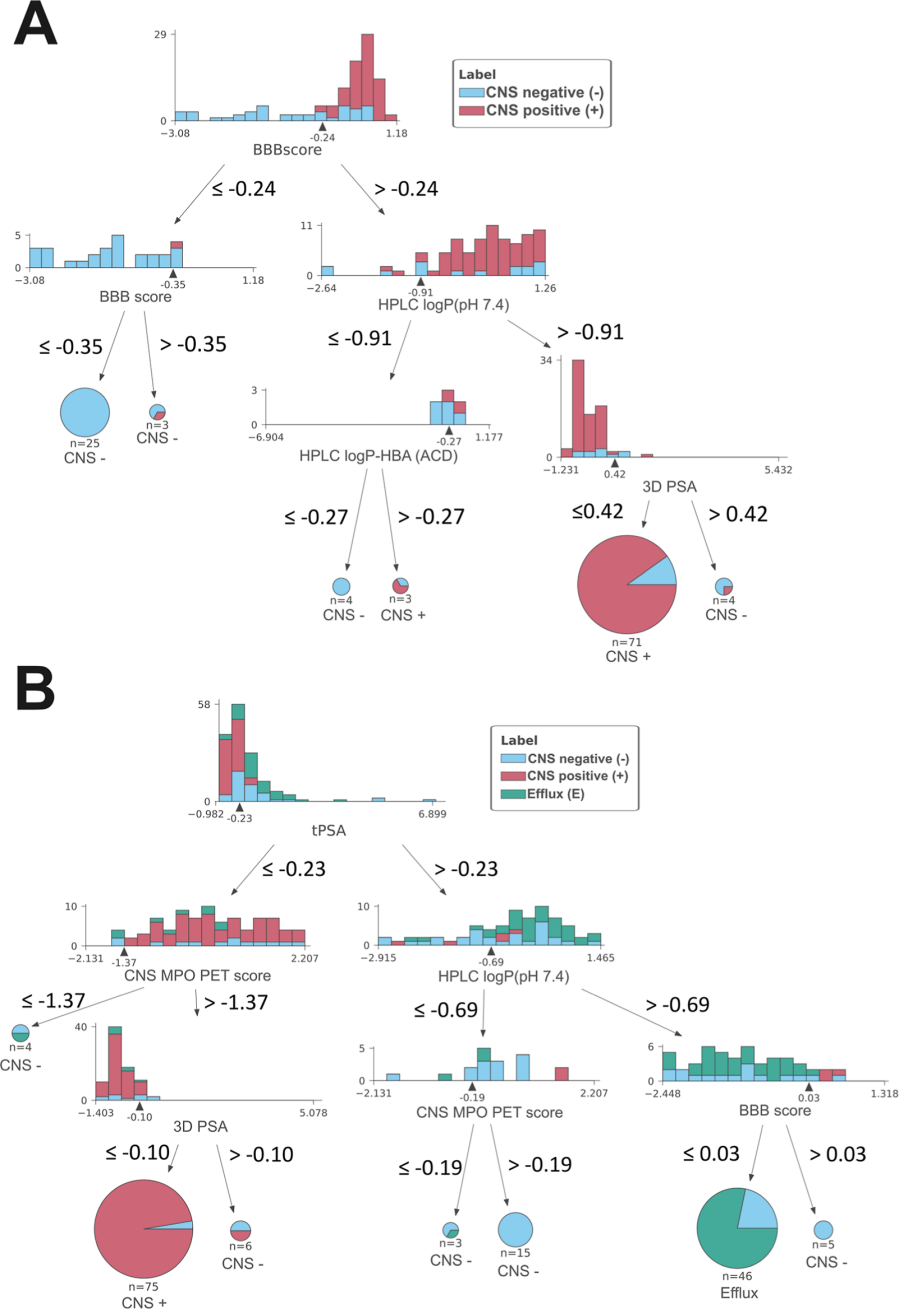
Approximation of the opaque random forest model using a simple decision tree model. (A) For the binary prediction and (B) for the multiclass prediction. To make a prediction for a molecule, the decision tree has to be followed from the top to the bottom until a leaf node (dead end) is reached. When a leaf node is met, the class is predicted, which is shown below the pie diagram. The pie diagrams and histogram show the distribution of classes expected at the position in the decision tree. All values are indicated in their standardized form.

For an explanation of the otherwise difficult-to-interpret random forest model, an approximation of the model was performed by predicting the output of the random forest model using a simple decision tree (Figure 5). Approximation performance was high, with ACC 0.85 (95% CI 0.83–0.87) and AUC 0.94 (95% CI 0.91–0.96). Surrogate modeling indicated that decision trees using only the features tPSA, 3D PSA, HPLC log *P*_ow_ (pH 7.4), and BBB score are sufficient for an estimation of the opaque model.

#### Comparative Assessment of Existing Scores

For the task of predicting CNS-negative, CNS-positive, and efflux transporter substrates, the traditional parameters CNS MPO, CNS MPO PET, and BBB score performed significantly worse compared with the ML-derived scoring. The CNS MPO score was associated with an AUC of 0.53, ACC 0.48, SNS 0.42, and SPC 0.64, while the CNS MPO PET score reached a similar AUC of 0.51 with ACC 0.44, SNS 0.42, and SPC 0.61. The BBB score showed slightly better performance with AUC 0.67, ACC 0.61, SNS 0.55, and SPC 0.82. In comparison, for the two-class classification only differentiating between CNS-negative and -positive compounds, performances for the existing scores were similar to the multiclass classification. While still performing substantially worse compared to the ML scoring, the BBB score was the best-performing parameter with AUC of 0.66, ACC of 0.66, SNS of 0.67, and SPC of 0.65. The CNS MPO score had AUC values of 0.54, ACC 0.57, SNS 0.60, and SPC 0.48. Despite achieving an AUC of 0.54, the accuracy indicated a close to random prediction for the CNS MPO PET scoring with ACC 0.49, SNS 0.44, and SPC 0.63. A comparison of all performance metrics of existing scores and the ML-derived score is shown in Table 2.

**Table 2:** Performance Comparison of Existing Scores for BBB Penetration with the Developed ML Model

		ACC	SNS	SPC	PPV	NPV	AUC
CNS MPO score	two-class	0.57	0.60	0.48	0.76	0.30	0.54
CNS MPO PET score	two-class	0.49	0.44	0.63	0.78	0.27	0.54
BBB score	two-class	0.66	0.67	0.65	0.84	0.41	0.66
**random forest model**	two-class	**0.83**	**0.88**	**0.75**	**0.87**	**0.79**	**0.88**
CNS MPO score	multiclass	0.48	0.42	0.64	0.30	0.30	0.53
CNS MPO PET score	multiclass	0.44	0.42	0.61	0.31	0.31	0.51
BBB score	multiclass	0.61	0.55	0.82	0.39	0.39	0.68
**random forest model**	**multiclass**	**0.67**	**0.62**	**0.82**	**0.63**	**0.63**	**0.82**

## Discussion and Conclusions

In this work, we have developed a novel three-dimensional calculation of the PSA value (3D PSA) and integrated it into a database containing molecular properties obtained from experimental and in silico methods, standardized, and referenced to the used software and method. This integration aims to improve the assessment of BBB permeability using ML. Further, we are providing the employed database for future calculations and comparisons, which includes 154 compounds and radiotracers, their ability to cross the BBB, or in vivo use as a CNS probe and the associated 24 features. To the best of our knowledge, this is the first database including 44 molecules including experimental values and categorized based on their interaction with efflux transporters, next to BBB penetration (passive diffusion, CNS active) and BBB nonpenetrating (CNS negative) compounds. The database primarily comprises radiotracers evaluated in in vivo studies. We demonstrated that this novel ML scoring approach outperforms the use of single molecular properties and evaluated three of the most common prediction approaches (BBB score, MPO CNS, and MPO CNS PET).

Since early drug discovery, the PSA was correlated with intestinal absorption,^34^ as well as BBB penetration, and, hence, was used for absorption, distribution, and BBB permeability models. Despite being a foundational parameter and useful in the prediction of the ability of a compound to cross the BBB, the PSA underlies substantial limitations. First, the PSA value is a relatively simple measure that considers only the polar surface in two dimensions, often differently calculated by various software packages and with difficulties in predicting unknown structures or molecules with higher MW. Second, most frequently used PSA predictions also do not consider optimized molecular geometry, as used in our 3D PSA calculations, which can lead to higher or lower solvent-accessible surface area inclusion and thus distort values. Lastly, PSA is often the first factor used for molecule description considering BBB penetration, or absorption, and is often used in isolation, without integrating it into broader predictive models due to time efficiency and the lack of other molecular parameters required to derive predictive scores. Together with the aforementioned limitations, this lack of contextual integration severely limits the PSA value’s utility in real-world drug discovery scenarios. In this work, we demonstrated a new in silico method for 3D PSA calculation. While still limited to some of the same factors as the (2D) PSA, this 3D PSA parameter takes the optimized geometry of molecules into account, which leads to a more precise surface area determination. The novel 3D PSA integrates atomic arrangement, molecular conformation, and surface topography like solvent-accessible pockets into polar surface calculations.

In the next step, we integrated the 3D PSA and conventional PSA with other molecular properties to allow for an integration that can balance the advantages and disadvantages of the individual parameters and combine their strengths to enable an optimal assessment of the ability of a compound to penetrate the BBB.

Our findings demonstrate a clear advantage of the ML scoring over existing individual molecular properties or predictive models. The 3D PSA value was among the most predictive features for the two-class model and an important parameter in both random forest models. Nevertheless, we observed that derivatives of the PSA, including the PSA (calculated by ACD), the tPSA, and the 3D PSA, consistently ranked among the most important single parameters for predicting BBB penetration in the ML analysis. Hence, taking into account the longer calculation time and higher computational power to calculate the 3D in comparison to the 2D models, the 3D calculation might be more useful in later stage, on respectively a smaller data set of candidates.

We further included the most often used predictive rules BBB score, CNS MPO, and CNS MPO PET in radiopharmaceutical development and included and validated them with our database and in our model. It is worth noting that the three scores were not primarily targeted toward predicting the same outcome as our model. While the BBB score infers BBB penetration via passive diffusion, the CNS MPO and CNS PET MPO scores further normalize to (in vitro) absorption, distribution, metabolism, excretion, and toxicity (ADME(T)) properties and provide an overall likelihood of success as a CNS drug or CNS PET tracer using the CNS PET MPO score and separate scores from ADME experiments, respectively. However, our database exceeds our simple classification as almost all positive compounds account for in vivo-established CNS PET tracers as well as our feature table includes experimental values, e.g., plasma protein binding with potentially more significance on pharmacokinetic influence than solely in silico values. Also in the comparative assessment, the BBB score performed the best, similar to the finding of Stéen et al.^35^ Overall, the BBB score achieved even better results than PSA-derived measures, being the most important feature in both the two-class and the multiclass prediction model.

The CNS MPO and CNS MPO PET were ranked generally worse on places 8 and 9 in the two-class model and 10 and 11th in the multiclass model. Important to note is that the experimental HPLC log *P*_owpH7.4_ was ranked in the middle field.

Next, we created a random forest model with an easy-to-interpret decision tree to approximate the opaque models two-class and multiclass model. Overall, for both models, the importance of the experimental value HPLC log *P*_ow_ph7.4_ is highlighted as well as the performance of the 3D PSA. In detail, the BBB score was in both models important to predict CNS-negative and efflux substrates, while HPLClog *P*_ow_ph7.4_, CNS MPO PET, and 3D PSA were the drivers for prediction of CNS-positive compounds. The ML model had a consistent tendency to predict higher probabilities for CNS-positive compounds when the 3D PSA was low.

Partition coefficient (log *P*) and distribution coefficient (log *D* (pH7.4)), two parameters that show the biggest variability, depending on the algorithm used, were not used for BBB score development; instead, they focused on HBD, HBA, and ionization constant (p*K*_a_) values that align more. The moderate importance of our analysis shows a moderate importance for the CNS MPO and CNS MPO PET score, which may be explained by its use of highly variable parameters, namely, Clog *P* and Clog *D* (pH 7.4). Even single parameters, like tPSA, PSA(ACD), HBD + HBA or p*K*_a_, show higher importance for BBB permeation prediction than the CNS MPO and CNS MPO PET scores because these values show less variation when calculated with different programs or using our standardized database. This highlights the importance of transparency and standardization when computing molecule properties.

The log *P* value despite being a subject of considerable debate remains a critical factor in CNS PET tracer development, particularly concerning absorption, distribution, unspecific binding, and metabolism. However, inconsistencies arise from the different methodologies used to calculate or measure log *P* values. Our database corroborates previous findings, suggesting that the majority of HPLC log *P*_ow_ (pH 7.4) and predicted Clog *P* values (e.g., those from ChemDraw) do not align, exhibiting significant variabilities across different calculation methods. This variability diminishes the reliability of log *P* as a predictive tool, as highlighted in other studies.^18,36,37^ The feature importance assessment further supports this, revealing that while the experimental HPLC log *P*_ow_ (pH 7.4) is among the most critical parameters, the in silico log *P* values, Clog *P* (ACD) and Clog *P* (ChemDraw), do not emerge as reasonably predictive. Zhang et al.^7,38^ demonstrated that combining experimental and predicted logarithms of brain/blood partitioning ratio at the steady state (log BB), a widely used parameter for BBB penetration, can improve accuracy. In the future, this approach could be implemented for our data set and tested for its predictive value. These findings emphasize the importance of experimental data for accurate predictions. However, it is important to note that even HPLC methods cannot be considered truly high-throughput as they require the synthesis of reference compounds. That said, HPLC is still more cost-effective, uses smaller amounts of compounds (only micrograms), and is faster than other experimental methods, such as cell culture models or artificial lipid bilayer experiments like the parallel artificial membrane permeability assay.^39^

Efflux transporters are a very heterogeneous class of molecules, often higher in MW than established CNS drugs and CNS PET tracer with complex pharmacokinetics and dynamics (concentration-dependent), which are known to be particularly difficult to predict. We assessed whether any of the scores used for comparison with the ML model were previously employed for the prediction of efflux transports. On 14 January 2025, we performed a search using the PubMed database for the query ((CNS MPO) AND (efflux transporter)) OR ((CNS MPO PET) AND (efflux transporter)) OR ((BBB score) AND (efflux transporter)). Among the five articles identified through this query, none investigated the prediction of efflux transporters using any of the three scores, indicating that this is the first study for assessing these scores’ ability to cross the BBB. Interestingly, despite the BBB score inherently predicting BBB permeability via passive diffusion, the BBB score performed best at identifying efflux transporter substrates among the three established scores. Still, the ML model, trained to predict efflux transports by integrating all three scores among other parameters, indicated a substantially better performance at predicting efflux transporter substrates.

In terms of practical implementation into research and drug development workflows, models based on the provided database may help researchers to prioritize compound testing, saving time and resources in experimental validation. The developed model further provides insights into the physicochemical properties driving BBB permeability, offering actionable guidance for rational drug design. The model’s prediction may be used to filter large compound libraries for BBB-permeable candidates, complementing existing experimental or computational approaches.

Since the usage of ML models built by other researchers can sometimes require substantial effort, computational knowledge, and sufficient details on the precise computational environment used, we decided to create surrogate models as part of this study. The surrogate decision tree models developed as part of this study provide an easy-to-follow visual representation that approximates the complex ML model and can be applied without using any computational knowledge or tools.

A strong asset of our data set lies in its heterogeneity, comprising a wide range of molecular structures, drug classes, or therapeutic indications. The standardization and transparency of the data, combined with the fact that most compounds have been successfully radiolabeled or are established pharmaceuticals, provide a solid foundation for reliable predictions. However, the results of this study must be interpreted in light of several limitations. Although the data set of 154 radiolabeled molecules (majority) provides a better ground truth compared to similar studies,^40−42^ it is still relatively small. The limited sample size may restrict the generalizability of the findings to broader drug discovery applications, particularly for molecules not represented within this data set. Nevertheless, the public availability of the data promotes transparency and the potential for further validation. The presented approach is partially based on the novel 3D PSA. While more precise, the calculations required to determine the 3D PSA are computationally intensive and, therefore, potentially limit fast high-throughput screening of large-scale databases. The study primarily relies on traditional in silico and experimental molecule properties for predicting BBB penetration. Although our ML approach improves predictive performance, it does not exploit the potential of deep learning techniques that may capture even more complex, nonlinear relationships between molecular features and BBB penetration or even PET CNS pharmacokinetics. Nevertheless, the approach proposed in this study represents are more interpretable solution while allowing for the future integration of deep learning models such as graph neural networks.^40^ While the study shows promising results for predicting BBB penetration, translating these findings into practical applications in drug development involves additional considerations. Incorporating pharmacokinetic values from dynamic PET-data such as the influx rate (*K*_1_) should be considered to improve the accuracy of the ground truth and the association of *K*_1_ and the predictive model or simpler approached as SUV or %ID cut-offs. However, this comes also with limitations of the scanner and PET protocol as well as species differences and is difficult to standardize. Further, the developed ML model does not explicitly account for pharmacokinetic properties. Instead, it is consideration for ADME processes results from its parameters, for example, via the integration of the CNS MPO score as predictor. Another limitation is the scarcity of radiolabeled CNS-negative compounds. In this study, the ground truth for negative (but not positive) BBB penetration was derived primarily from well-established drugs with known kinetics or preclinical models rather than human PET studies.

While radiolabeling of new drug candidates for clinical phase studies are common,^43^ initiatives such as “eatris”^44^ promote using functional imaging for drug development. However, to the best of our knowledge, negative results in radiotracer and drug development with regard to the BBB penetration are rarely shared. Recently, the importance and need for large, well-characterized data sets, including solid ground truth assessments, sharing of negative results, and the importance of computational prediction models for successful PET CNS tracer are increasingly highlighted.^45,46^ With this work, we aim to encourage the scientific community involved in (CNS) PET tracer development to share their preclinical PET data of compounds that do not penetrate the BBB, therefore contributing to the refinement of predictive models.

This study highlights the potential of advancing BBB penetration prediction, which is crucial for CNS drug development with a standardized and transparent database. Employing explainable ML, integrating in vivo measurements on BBB penetration of radiolabeled molecules and molecular parameters derived using various experimental and in silico methods, we demonstrate enhanced predictive accuracy over the traditional concept of applying individual molecular properties. Our findings highlight the superiority of ML models compared with conventional scores in determining molecule permeability through the BBB. This approach promises to contribute to refining and expediting the CNS drug development process, reducing the need for animal studies.

## Data Availability

The full list of all 154 molecules, including their physicochemical parameters and other molecular properties and the associated information on in vivo permeability of the BBB, is publicly accessible via https://osf.io/cvhe9. We encourage researchers to share their data to extend this database. All employed software components are open source. Please refer to the sections “Machine learning” and “Statistical analysis” for the names of individual software packages.

## Supporting Information Available

Additional analysis results, materials, and methodological details (PDF)
