# Supplementary material for: Enhancing Blood–Brain Barrier Penetration Prediction by Machine Learning-Based Integration of Novel and Existing, In Silico and Experimental Molecular Parameters from a Standardized Database
Source: J Chem Inf Model. 2025 Mar 4;65(6):2773–84. doi: 10.1021/acs.jcim.4c02212 (PMC11938273; doi:10.1021/acs.jcim.4c02212)
Supplement: Supplementary file 1 — ci4c02212_si_001.pdf [file ci4c02212_si_001.pdf]

# SUPPORTING INFORMATION

# Enhancing blood-brain barrier penetration prediction by machine learning-based integration of novel and existing, *in silico* and experimental molecular parameters from a standardized database

*Clemens P. Spielvogel*[1,2], Natalie Schindler*[1], Christian Schröder[3], Sarah Luise Stellnberger[5], Wolfgang Wadsak[1,6], Markus Mitterhauser[1,7, 9], Laszlo Papp[8], Marcus Hacker[1], Verena Pichler[4], and Chrysoula Vraka[1]*

**First authors contributed equally*

[1] Division of Nuclear Medicine, Department of Biomedical Imaging and Image-Guided Therapy, Medical University of Vienna, Vienna, Austria

[2] Christian Doppler Laboratory for Applied Metabolomics, Vienna, Austria.

[3] Department of Computational Biological Chemistry, University of Vienna, Vienna, Austria.[4] Department of Pharmaceutical Sciences, Division of Pharmaceutical Chemistry, University of Vienna, Vienna Austria.

[4] Division of Pharmaceutical Chemistry, Department of Pharmaceutical Sciences, Faculty of Life Sciences, University of Vienna, Josef-Holaubek-Platz 2, 1090 Vienna, Austria

[5] Vienna Doctoral School of Pharmaceutical, Nutritional and Sport Sciences, University of Vienna, Josef-Holaubek-Platz 2, 1090 Vienna, Austria

[6] MINUTE medical GmbH, Vienna, Austria

[7] Joint Applied Medicinal Radiochemistry Facility of the University of Vienna and the Medical University of Vienna, Vienna, Austria

[8] Center for Medical Physics and Biomedical Engineering, Medical University of Vienna, Vienna, Austria

[9] Institute of Inorganic Chemistry, Faculty of Chemistry, Währinger Strasse 42, 1090 Vienna, Austria

Number of pages: 37

Number of figures: 2

Number of tables: 1

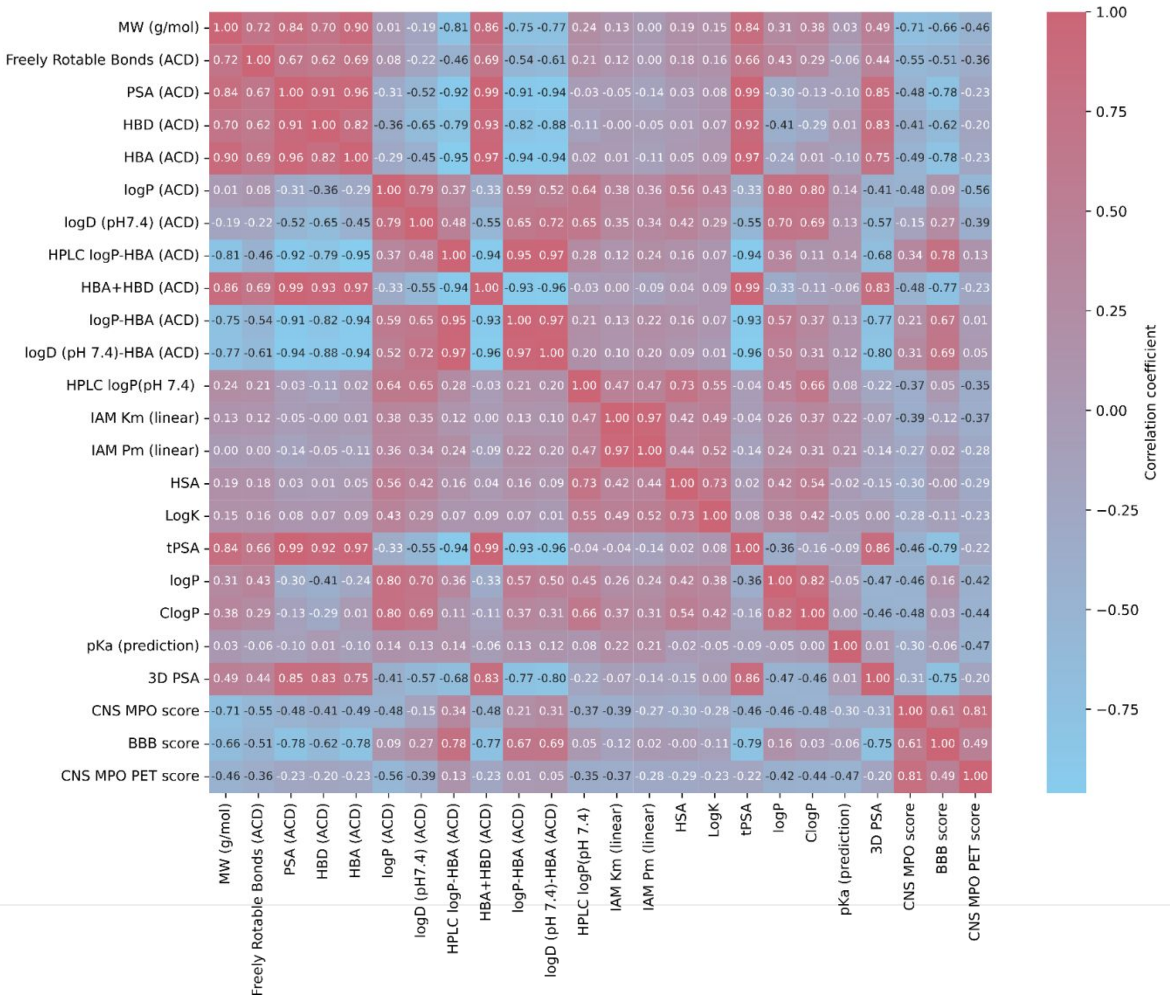


x

**Figure S1.** Correlation of the 24 employed parameters.

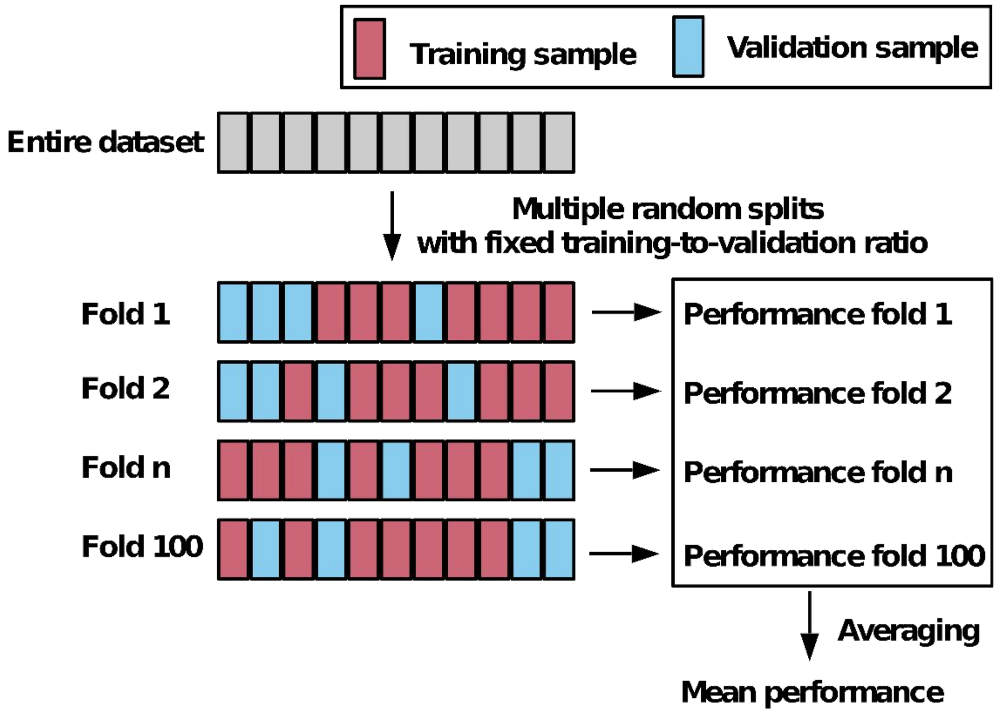


**Figure S2.** Monte Carlo cross-validation scheme. Procedure for the performance measurement of machine learning models. The entire data is split multiple times with a given training-to-validation ratio. The training samples in each split are used to train a machine learning model. The performance of this model is assessed using the corresponding validation data. In this way, each fold (train-validation-split) yields a performance (e.g. accuracy). The performances are then averaged using the mean to yield a final performance.

**Compounds**

**Table S1.** List of all employed compounds and associated CNS status. A positive CNS status indicates BBB penetration.

| Compound name | IUPAC name | CNS status | Radiolabeled | SMILES |
|---|---|---|---|---|
| FE@SNAP | (2-[18F]fluoroethyl)3-((3-(4-(3-acetamidophenyl)piperidin-1-yl)propyl)carbamoyl)-4-(3,4-difluorophenyl)-6-(methoxymethyl)-2-oxo-1,2,3,4-tetrahydropyrimidine-5-carboxylate | Efflux transporter substrate | [1,2] | O=C(OCCF)C1=C(COC)NC(=O)N(C1c1ccc(F)c(F)c1)C(=O)NCCCN1CCC(CC1)c1cccc(NC(C)=O)c1 |

| | | | | |
|---|---|---|---|---|
| FE@SUPPY | 5-(2-[18F]fluoroethyl)2,4-diethyl-3-(ethylsulfanylcarbonyl)-6-phenylpyridine-5-carboxylate | Negative | [3,4] | CCc1nc(c(c(CC)c1C(=O)SCC)C(=O)OCC[18F])c1ccccc1 |
| 2-FE-beta-CIT | (1R,2S,3S,5S)-2-fluoroethyl-3-(4-iodophenyl)-8-methyl-8-azabicyclo[3.2.1]octane-2-carboxylate | Positive | [5,6] | CN1[C@H]2CC[C@@H]1[C@H]([C@H](C2)c1ccc(I)cc1)C(=O)OCCF |
| 6-meO-BTA-0 | 2-(4-(Methylamino)phenyl)benzo[d]thiazol-6-ol | Negative | | Nc1ccc(cc1)c1nc2ccc(cc2s1)OC |
| 6-OH-BTA-1 (PiB) | 2-(4-(Methylamino)phenyl)benzo[d]thiazol-6-ol | Positive | [7,8] | CNc1ccc(cc1)c1nc2ccc(O)cc2s1 |
| ABP688 | (E)-3-((6-methylpyridin-2-yl)ethynyl)cyclohex-3- enone O-methyl oxime | Positive | [9,10] | Cc1cccc(C#CC2=CCC/C(=N\OC)C2)n1 |
| Actinomycin D | 2-AminoN,N′-bis[(6S,9R,10S,13R,18aS)-6,13-diisopropyl-2,5,9-trimethyl-1,4,7,11,14-pentaoxohexadecahydro-1H-pyrrolo[2,1-i][1,4,7,10,13]oxatetraazacyclohexadecin-10-yl]-4,6-dimethyl-3-oxo-3H-phenoxazine-1,9-dicarboxamide 2-amino-4,6-dimethyl-3-oxo-1-*N*,9-*N*-bis[(3*R*,6*S*,7*R*,10*S*,16*S*)-7,11,14-trimethyl-2,5,9,12,15-pentaoxo-3,10-di(propan-2-yl)-8-oxa-1,4,11,14-tetrazabicyclo[14.3.0]nonadecan-6-yl]phenoxazine-1,9-dicarboxamide | Efflux transporter substrate | | CC(C)[C@@H]1NC(=O)[C@H](NC(=O)c2ccc(C)c3OC4=C(C)C(=O)C(N)=C(C4=Nc23)C(=O)N[C@H]2C(=O)N[C@H](C(=O)N3CCC[C@@H]3C(=O)N(C)CC(=O)N(C)[C@H](C(C)C)C(=O)O[C@H]2C)C(C)C)[C@H](C)OC(=O)[C@@H](C(C)C)N(C)C(=O)CN(C)C(=O)[C@H]2CCCN2C1=O |
| Altanserin | 3-[2-[4-(4-fluorobenzoyl)piperidin-1-yl]ethyl]-2-sulfanylidene-1*H*-quinazolin-4-one | Positive | [11,12] | Fc1ccc(cc1)C(=O)C1CCN(CCN2C(=O)c3ccccc3NC2=S)CC1 |
| Amprenavir | [1,4,7,10,13]oxatetraazacyclohexadecin-10-yl]-4,6-dimethyl-3-oxo-3H-phenoxazine-1,9-dicarboxamide | Efflux transporter substrate | | O=C(N[C@@H](Cc1ccccc1)[C@H](O)CN(CC(C)C)S(=O)(=O)c1ccc(N)cc1)O[C@H]1CCOC1 |

| | | | | |
|---|---|---|---|---|
| Asimadoline | N-{(1S)-2-[(3S)-3-Hydroxy-1-pyrrolidinyl]-1-phenylethyl}- Nmethyl-2,2-diphenylacetamide | Efflux transporter substrate | | CN(C(CN1CC[C@H](O)C1)c1ccccc1)C(=O)C(c1ccccc1)c1ccccc1 |
| Atorvastatin | (3R,5R)-7-[2-(4-Fluorophenyl)-5-isopropyl-3-phenyl-4-(phenylcarbamoyl)-1H-pyrrol-1-yl]-3,5-dihydroxyheptanoic acid | Efflux transporter substrate | | O[C@H](CCn1c(c(c(c1C(C)C)C(=O)Nc1ccccc1)c1ccccc1)c1ccc(F)cc1)C[C@@H](O)CC(=O)O |
| Azidomazenil | Ethyl-8-azido-5-methyl-6-oxo-4*H*-imidazo[1,5-a][1,4]benzodiazepine-3-carboxylate | Positive | 13 | O=C(OCC)c1ncn2c1CN(C)C(=O)c1cc(/N=[N+]=[N-])ccc12 |
| beta-CFT | (1R,2S,3S,5S)-methyl-3-(4-fluorophenyl)-8-((E)-3-iodoallyl)- 8-azabicyclo[3.2.1]octane-2-carboxylate | Positive | 6,14 | CN1[C@H]2CC[C@@H]1[C@H]([C@H](C2)c1ccc(F)cc1)C(=O)OC |
| beta-CIT | (1R,2S,3S,5S)-methyl-3-(4-iodophenyl)-8-methyl-8-azabicyclo [3.2.1]octane-2-carboxylate | Positive | 15 | CN1[C@H]2CC[C@@H]1[C@H]([C@H](C2)c1ccc(I)cc1)C(=O)OC |
| beta-CIT-FP | rac-methyl(1R,2S,3S,5S)-8-(3-fluoropropyl)-3-(4-iodophenyl)-8-azabicyclo[3.2.1]octane-2-carboxylate | Positive | 15 | Ic1ccc(cc1)[C@H]1C[C@@H]2CC[C@H]([C@H]1C(=O)OC)N2CCCF |
| beta-CPPIT | 5-((1R,2S,3S,5S)-3-(4-chlorophenyl)-8-methyl-8-azabicyclo[3.2.1]octan-2-yl)-3-phenylisoxazole | Positive | 16 | Clc1ccc(cc1)C1CC2CCC(N2C)C1c1cc(no1)c1ccccc1 |
| Bisantrene | *N*-[(*E*)-[10-[(*E*)-(4,5-dihydro-1*H*-imidazol-2-ylhydrazinylidene)methyl]anthracen-9-yl]methylideneamino]-4,5-dihydro-1*H*-imidazol-2-amine | Efflux transporter substrate | | N(N\C=C1\NCCN1)c1c2ccccc2c(NN\C=C2/NCCN2)c2ccccc12 |
| BMP | 1-methylpiperidin-4-yl butyrate | Positive | 17 | CN1CCC(OC(=O)CCC)CC1 |

| | | | | |
|---|---|---|---|---|
| Buprenorphine | rac-(4R,4aS,6R,7R,7aR,12bS)-3-(cyclopropylmethyl)-6-((S)-2-hydroxy-3,3-dimethylbutan-2-yl)-7-methoxy-1,2,3,4,5,6,7,7a-octahydro-4a,7-ethano-4,12-methanobenzofuro[3,2-e]isoquinolin-9-ol | Positive | | CC(C)(C)[C@@](C)(O)[C@H]1C[C@]23CC[C@]1(OC)[C@@H]1Oc4c(O)ccc5C[C@H]2N(CC[C@]13c54)CC1CC1 |
| Candesartan | 1-((2'-(2H-tetrazol-5-yl)-[1,1'-biphenyl]-4-yl)methyl)-2-ethoxy-1H-benzo[d]imidazole-7-carboxylic acid | Negative | | O=C(O)c1cccc2nc(OCC)n(Cc3ccc(cc3)c3ccccc3c3n[NH]nn3)c12 |
| Candesartan cilexetil | 1-{[(Cyclohexyloxy)carbonyl]oxy}ethyl 2-ethoxy-1-{[2′-(1H-tetrazol-5-yl)- 4-biphenylyl]methyl}-1Hbenzimidazole-7-carboxylate | Negative | | O=C(OC1CCCCC1)OC(C)OC(=O)c1cccc2nc(OCC)n(Cc3ccc(cc3)c3ccccc3c3nnn[NH]3)c12 |
| Carazolol | 1-((9H-carbazol-4-yl)oxy)-3-(isopropylamino)propan-2-ol | Positive | [18,19] | CC(C)NCC(O)COc1cccc2[NH]c3ccccc3c12 |
| Carbidopa | (2S)-3-(3,4-Dihydroxyphenyl)-2-hydrazino-2-methylpropanoic acid | Negative | | Oc1ccc(C[C@](C)(NN)C(=O)O)cc1O |
| Carfentanil oxalat | Methyl 1-phenethyl-4-(N-phenylpropionamido) piperidine-4-carboxylate | Positive | [20,21] | O=C(OC)C1(CCN(CCc2ccccc2)CC1)N(c1ccccc1)C(=O)CC |
| Celecoxib | 4-[5-(4-Methylphenyl)-3-(trifluoromethyl)-1H-pyrazol-1-yl]benzenesulfonamide | Negative | | NS(=O)(=O)c1ccc(cc1)n1nc(cc1c1ccc(C)cc1)C(F)(F)F |
| Cetirizin | (2-{4-[(4-Chlorophenyl)(phenyl)methyl]-1-piperazinyl}ethoxy)acetic acid | Negative | | O=C(O)COCCN1CCN(CC1)C(c1ccc(Cl)cc1)c1ccccc1 |
| Cimetidine | 1-Cyano-2-methyl-3-(2-{[(4-methyl-1H-imidazol5-yl)methyl]sulfanyl}ethyl)guanidine | Efflux transporter substrate | | Cc1nc[NH]c1CSCCNC(=N\C)\NC#N |

| | | | | |
|---|---|---|---|---|
| CIT-FE | (1R,2S,3S,5S)-methyl 8-(2-fluoroethyl)-3-(4-iodophenyl)-8- azabicyclo[3.2.1]octane-2-carboxylate | Positive | [6,22] | Ic1ccc(cc1)[C@H]1C[C@@H]2CC[C@H]([C@H]1C(=O)OC)N2CCF |
| CITFES | 8-Azabicyclo[3.2.1]octane-2-carboxylic acid | Positive | | Ic1ccc(cc1)C1CC2CCC(C1C(=O)O)N2CCF |
| CITFP | 8-(3-fluoropropyl)-3-(4-iodophenyl)-, methyl ester, (1R,2S,3S,5S)- | Positive | [23,24] | COC(=O)C1C2CCC(N2CCCF)CC1C3=CC=C(C=C3)I |
| Clarithromycin | rac-(3R,4S,5S,6R,7R,9R,11R,12R,13S,14R)-6-(((2S,3R,4S,6R)-4-(dimethylamino)-3-hydroxy-6-methyltetrahydro-2H-pyran-2-yl)oxy)-14-ethyl-12,13-dihydroxy-4-(((2R,4R,5S,6S)-5-hydroxy-4-methoxy-4,6-dimethyltetrahydro-2H-pyran-2-yl)oxy)-7-methoxy-3,5,7,9,11,13-hexamethyloxacyclotetradecane-2,10-dione | Negative | | CN(C)[C@H]1C[C@@H](C)O[C@@H](O[C@@H]2[C@@H](C)[C@H](O[C@H]3C[C@@](C)(OC)[C@@H](O)[C@H](C)O3)[C@@H](C)C(=O)O[C@H](CC)[C@@](C)(O)[C@H](O)[C@@H](C)C(=O)[C@H](C)C[C@@]2(C)OC)[C@@H]1O |
| Clindamycin | rac-(1R,4S)-N-((1R)-2-chloro-1-((2S,3R,4S,5R,6R)-3,4,5-trihydroxy-6-(methylthio) tetrahydro-2H-pyran-2-yl)propyl)-2-methyl-4-propylcyclopentane-1-carboxamide | Negative | | O[C@@H]1[C@@H](O)[C@@H](O)[C@@H](O[C@@H]1SC)[C@@H](NC(=O)[C@@H]1C[C@@H](CCC)CC1C)C(C)Cl |
| Colchicine | (S,E)-N-(1,2,3,10-tetramethoxy-9-oxo-5,6,7,9-tetrahydrobenzo[a]heptalen-7-yl)acetimidic acid | Efflux transporter substrate | | C\C(O)=N/[C@H]1CCc2cc(OC)c(OC)c(OC)c2C2=CC=C(OC)C(=O)C=C21 |
| Colistin | rac-N-((R)-4-amino-1-(((2R,3S)-1-(((R)-4-amino-1-oxo-1-(((3S,6R,9R,12R,15S,18R,21R)-6,9,18-tris(2-aminoethyl)-3-((S)-1-hydroxyethyl)-12,15-diisobutyl-2,5,8,11,14,17,20-heptaoxo-1,4,7,10,13,16,19-heptaazacyclotricosan-21-yl)amino)butan-2-yl)amino)-3-hydroxy-1-oxobutan-2-yl)amino)-1-oxobutan-2-yl)-5-methylheptanamide | Negative | | CC(CC)CCCC(=O)N[C@@H](CCN)C(=O)N[C@@H]([C@@H](C)O)C(=O)N[C@@H](CCN)C(=O)N[C@H]1CCNC(=O)[C@@H](NC(=O)[C@H](CCN)NC(=O)[C@H](CCN)NC(=O)[C@H](CC(C)C)NC(=O)[C@@H](CC(C)C)NC(=O)[C@@H](NC1=O)CCN)[C@@H](C)O |
| Corticosterone | (11β)-11,21-Dihydroxypregn-4-ene-3,20-dione | Efflux transporter substrate | | OCC(=O)[C@H]1CC[C@H]2[C@@H]3CCC4=CC(=O)CC[C@]4(C)[C@H]3[C@@H](O)C[C@]12C |

| | | | | |
|---|---|---|---|---|
| CPFPX | 8-Cyclopentyl-3-(3-fluoropropyl)-1-propyl-1H-purine-2,6(3H,7H)-dione | Positive | [25–27] | O=C1c2[NH]c(nc2N(CCCF)C(=O)N1CCC)C1CCCC1 |
| Cyclosporin A | (3S,6S,9S,12R,15S,18S,21S,24S,30S,33S)-30-Ethyl-33-[(1R,2R,4E)-1-hydroxy2-methyl-4-hexen-1-yl]-6,9,18,24-tetraisobutyl-3,21-diisopropyl1,4,7,10,12,15,19,25,28-nonamethyl-1,4,7,10,13,16,19,22,25,28,3 1-undecaazacyclotritri acontane-2,5,8,11,14,17,20,23,26,29,32-undecone | Efflux transporter substrate | | C[C@H](C\C=C\C)[C@@H](O)[C@H]1C(=O)N[C@@H](CC)C(=O)N(C)CC(=C)N(C)[C@@H](CC(C)C)C(=O)N[C@@H](C(C)C)C(=O)N(C)[C@@H](CC(C)C)C(=O)N[C@@H](C)C(=O)N[C@H](C)C(=O)N(C)[C@@H](CC(C)C)C(=O)N(C)[C@@H](CC(C)C)C(=O)N(C)[C@@H](C(C)C)C(=O)N1C |
| DAA1106 | N-(2,5-dimethoxybenzyl)-N-(5-fluoro-2-phenoxyphenyl)acetamide | Positive | [28,29] | CC(=O)N(Cc1cc(OC)ccc1OC)c1cc(F)ccc1Oc1ccccc1 |
| DASB | 3-Amino-4-((2-((dimethylamino)methyl)phenyl)thio)benzonitrile | Positive | [30,31] | CN(C)Cc1ccccc1Sc1ccc(cc1N)C#N |
| Daunorubicin | (1S,3S)-3-Acetyl-3,5,12-trihydroxy-10-methoxy-6,11-dioxo-1,2,3,4,6,11-hexahydro-1-tetracenyl 3-amino-2,3,6-trideoxy-α-L-lyxo-hexopyranoside | Efflux transporter substrate | | CC(=O)[C@@]1(O)C[C@H](O[C@H]2C[C@H](N)[C@H](O)[C@H](C)O2)c2c(O)c3C(=O)c4c(OC)cccc4C(=O)c3c(O)c2C1 |
| Dexamethason | (11β,16α)-9-Fluoro-11,17,21-trihydroxy-16-methylpregna-1,4-diene-3,20-dione | Efflux transporter substrate | | OCC(=O)[C@@]1(O)[C@@H](C)C[C@H]2[C@@H]3CCC4=CC(=O)C=C[C@]4(C)C3(F)[C@H](O)C[C@@]21C |
| Dexrazoxane | rac-(R)-4,4'-(propane-1,2-diyl)bis(piperazine-2,6-dione) | Negative | | O=C1CN(C[C@H](C)N2CC(=O)NC(=O)C2)CC(=O)N1 |

| | | | | |
|---|---|---|---|---|
| Digoxin | (3β,5β,12β)-3-{[2,6-Dideoxy-β-D-ribo-hexopyranosyl-(1-N;4)-2,6-dideoxyβ-D-ribo-hexopyranosyl-(1-N4)-2,6-dideoxy-β-D- ribo-hexopyranosyl]oxy}- 12,14-dihydroxycard-20(22)-enolide | Efflux transporter substrate | | O=C1C=C(CO1)[C@H]1CC[C@]2(O)[C@@H]3CC[C@@H]4C[C@H](CC[C@]4(C)[C@H]3C[C@@H](O)[C@]12C)O[C@@H]1O[C@H](C)[C@@H](O[C@H]2C[C@H](O)[C@H](O[C@H]3C[C@H](O)[C@H](O)[C@@H](C)O3)[C@@H](C)O2)[C@@H](O)C1 |
| Diltiazem | (2S,3S)-5-[2-(Dimethylamino)ethyl]-2-(4-methoxyphenyl)-4-oxo-2,3,4,5- tetrahydro-1,5-benzothiazepin-3-yl acetate | Efflux transporter substrate | | CN(C)CCN1c2ccccc2S[C@H]([C@@H](OC(C)=O)C1=O)c1ccc(OC)cc1 |
| Diprenorphine | rac-(4aR,6S,7S,7aS,12bR)-3-(cyclopropylmethyl)-6-(2-hydroxypropan-2-yl)-7-methoxy-1,2,3,4,5,6,7,7a-octahydro-4a,7-ethano-4,12-methanobenzofuro[3,2-e]isoquinolin-9-ol | Positive | 32–34 | CC(C)(O)[C@H]1C[C@]23CC[C@]1(OC)[C@@H]1Oc4c(O)ccc5CC2[N@@](CC[C@]13c54)CC1CC1 |
| DMFEAN | 2-(1-(6-((2-Fluoroethyl)(methyl)amino)naphthalen2-yl)ethylidene)malononitrile | Positive | | N#C/C(C#N)=C(\C)c1ccc2cc(ccc2c1)N(C)CCF |
| Domperidon | 5-Chloro-1-{1-[3-(2-hydroxy-1Hbenzimidazol-1-yl)propyl]-4-piperidinyl}- 1H-benzimidazol-2-ol | Negative | | Clc1cc2nc(O)n(C3CCN(CCCn4c5ccccc5nc4O)CC3)c2cc1 |
| Doxorubicin | (1S,3S)-3-Glycoloyl-3,5,12-trihydroxy-10-methoxy-6,11-dioxo-1,2,3,4,6,11-hexahydro-1-tetracenyl 3-amino-2,3,6-trideoxy-α-L-lyxo-hexopyranoside | Efflux transporter substrate | | OCC(=O)[C@@]1(O)C[C@H](O[C@H]2C[C@H](N)[C@H](O)[C@H](C)O2)c2c(O)c3C(=O)c4c(OC)cccc4C(=O)c3c(O)c2C1 |
| Elacridar | N-{4-[2-(6,7-Dimethoxy-3,4-dihydro-2(1H)-isoquinolinyl)ethyl]phenyl}- 5-methoxy-9-oxo-9,10-dihydro-4-acridinecarboxamide | Efflux transporter substrate | 35,36 | O=C1c2cccc(c2Nc2c1cccc2OC)C(=O)Nc1ccc(cc1)CCN1Cc2cc(OC)c(cc2CC1)OC |
| Entacapone | (2E)-2-Cyano-3-(3,4-dihydroxy-5-nitrophenyl)-N,N-diethylacrylamide | Negative | | Oc1cc(\C=C(/C#N)C(=O)N(CC)CC)cc([N+]([O-])=O)c1O |

| | | | | |
|---|---|---|---|---|
| Epidepride | (S)-N-((1-ethylpyrrolidin-2-yl)methyl)-5-iodo-2,3-dimethoxybenzamide | Positive | [37,38] | CCN1CCC[C@H]1CNC(=O)c1cc(I)cc(OC)c1OC |
| Epirubicin | (1S,3S)-3-Glycoloyl-3,5,12-trihydroxy-10-methoxy-6,11-dioxo-1,2,3,4,6,11-hexahydro-1-tetracenyl 3-amino-2,3,6-trideoxy-α-L-arabino-hexopyranoside | Efflux transporter substrate | | OCC(=O)[C@@]1(O)C[C@H](O[C@H]2C[C@H](N)[C@@H](O)[C@H](C)O2)c2c(O)c3C(=O)c4c(OC)cccc4C(=O)c3c(O)c2C1 |
| Erlotinib | N-(3-Ethynylphenyl)-6,7-bis(2-methoxyethoxy)-4-quinazolinamine | Efflux transporter substrate | [39] | C#Cc1cc(ccc1)Nc1ncnc2cc(OCCOC)c(cc21)OCCOC |
| Etomidate | (R)-ethyl 1-(1-phenylethyl)-1H-imidazole-5-carboxylate | Positive | | C[C@H](c1ccccc1)n1cncc1C(=O)OCC |
| Etoposide | (5S,5aR,8aR,9R)-9-(4-Hydroxy-3,5-dimethoxyphenyl)-8-oxo-5,5a,6,8,8a,9-hexahydrofuro[3′,4′:6,7]naphtho[2,3-d][1,3]dioxol-5-yl 4,6-O-[(1R)-ethylidene]-β-D-glucopyranoside | Efflux transporter substrate | | COc1cc(cc(OC)c1O)[C@H]1c2cc3OCOc3cc2[C@H](O[C@H]2O[C@H]3CO[C@H](C)O[C@@H]3[C@@H](O)[C@@H]2O)[C@@H]2COC(=O)[C@@H]12 |
| Fallypride | (S)-N-((1-allylpyrrolidin-2-yl)methyl)-1-(5-(3-fluoropropyl)- 2,3-dimethoxyphenyl)ethenamine | Positive | | C=CCN1CCC[C@H]1CNC(=O)c1cc(CCCF)cc(OC)c1OC |
| FE-Carfentanil | 2-fluoroethyl 1-phenethyl-4-(Nphenylpropionamido)piperidine-4-carboxylate | Positive | [40] | O=C(OCCF)C1(CCN(CCc2ccccc2)CC1)N(c1ccccc1)C(=O)CC |
| FE-IPCIT | (1R,2S,3S,5S)-2-fluoroethyl 8-((E)-3-iodoallyl)-3-(4-iodophenyl)- 8-azabicyclo[3.2.1]octane-2-carboxylate | Positive | [15] | Ic1ccc(cc1)C1CC2CCC(C1C(=O)OCCF)N2C\C=C\I |
| FE-PE2i | 2-fluoroethyl (1R,2S,3S,5S)-8-((E)-3-iodoallyl)-3-(p-tolyl)-8-azabicyclo[3.2.1]octane-2-carboxylate | Positive | [41,42] | Cc1ccc(cc1)C1CC2CCC(C1C(=O)OCCF)N2C\C=C\I |

| | | | | |
|---|---|---|---|---|
| FETA | N-(2-fluoroethyl)-2-(2-nitro-1H-imidazol-1-yl)acetamide | Positive | 43 | O=C(Cn1ccnc1[N+]([O-])=O)NCCF |
| Fexofenadine | 2-[4-(1-Hydroxy-4-{4-[hydroxy(diphenyl)methyl]-1-piperidinyl}butyl)phenyl]- 2-methylpropanoic acid | Efflux transporter substrate | | O=C(O)C(C)(C)c1ccc(cc1)C(O)CCCN1CCC(CC1)C(O)(c1ccccc1)c1ccccc1 |
| FLB 457 | (S)-5-Bromo-N-((1-ethylpyrrolidin-2-yl)methyl)-2,3- dimethoxybenzamide | Positive | 44,45 | CCN1CCC[C@H]1CNC(=O)c1cc(Br)cc(OC)c1OC |
| Flumazenil | Ethyl 8-fluoro5-methyl-6-oxo-5,6-dihydro-4H-benzo[f]imidazo[1,5-a] [1, 4]diazepine-3-carboxylate | Positive | 46–48 | O=C(OCC)c1ncn2c1CN(C)C(=O)c1cc(F)ccc12 |
| Fluoroethyl Etomidate (FETO) | (R)-2-fluoroethyl 1-(1-phenylethyl)-1H-imidazole-5-carboxylate | Positive | 49 | C[C@H](c1ccccc1)n1cncc1C(=O)OCCF |
| Fluoroethyl-Carfentanil hydrochloride | 2-fluoroethyl 4-[N-(1-oxopropyl)-N-phenylamino]-1-(2-phenylethyl)-4-piperidinecarboxylate | Positive | 40 | O=C(OCCF)C1(CCN(CCc2ccccc2)CC1)N(c1ccccc1)C(=O)CC |
| Fluoroethylflumazenil (FFMZ) | 2-Fluoroethyl 8-fluoro-5-methyl-6-oxo-5,6-dihydro-4H-benzo [f]imidazo[1,5-a] [1, 4]diazepine-3-carboxylate | Positive | 50,51 | O=C(OCCF)c1ncn2c1CN(C)C(=O)c1cc(F)ccc12 |
| Fluoroethylspiperon (FESP) | 3-(2-Fluoroethyl)-8-(4-(4-fluorophenyl)-4-oxobutyl)-1-phenyl1,3,8-triazaspiro[4.5]decan-4-one | Positive | 52–54 | Fc1ccc(cc1)C(=O)CCCN1CCC2(CC1)C(=O)N(CN2c1ccccc1)CCF |
| FluoromethylMcN 5652 | (6S,10bR)-6-(4-((fluoromethyl)thio)phenyl)-1,2,3,5,6,10bhexahydropyrrolo[2,1-a]isoquinoline | Positive | 55,56 | FCSc1ccc(cc1)[C@@H]1CN2CCCC2c2ccccc21 |

| | | | | |
|---|---|---|---|---|
| Fluoromisonidazole (FMISO) | 1-Fluoro-3-(2-nitro-1H-imidazol-1-yl)propan-2-ol | Positive | [57] | OC(Cn1ccnc1[N+]([O-])=O)CF |
| Flutemetamol | 2-(3-fluoro-4-(methylamino)phenyl)benzo[d]thiazol-6-ol | Positive | [58] | CNc1ccc(cc1F)c1nc2ccc(O)cc2s1 |
| FNMB | 1-(1-(4-(4-Fluorophenyl)-4-oxobutyl)piperidin-4-yl)-3-methyl1H-benzo[d]imidazol-2(3H)-one | Positive | [59] | Fc1ccc(cc1)C(=O)CCCN1CCC(CC1)N1c2ccccc2N(C)C1=O |
| FPEB | 3-Fluoro-5-(pyridin-2-ylethynyl)benzonitrile | Positive | [60] | N#Cc1cc(C#Cc2ccccn2)cc(F)c1 |
| Fudinsäure | (3α,4α,5α,8α,9β,11α,13α,14β,16β,17Z)- 16-Acetoxy-3,11-dihydroxy-4,8,14- trimethyl-18-norcholesta-17,24-dien- 21-oic acid | Negative | | O=C(O)C(\CC\C=C(/C)C)=C1\[C@@H]2C[C@@H](O)[C@H]3[C@@]4(C)CC[C@@H](O)[C@@H](C)[C@@H]4CC[C@]3(C)[C@@]2(C)C[C@@H]1OC(C)=O |
| Fulvestrant | (7α,17β)-7-{9-[(4,4,5,5,5-Pentafluoropentyl)sulfinyl]nonyl}estra-1(10),2, 4-triene-3,17-diol | Negative | | FC(F)(F)C(F)(F)CCCS(=O)CCCCCCCCC[C@@H]1Cc2cc(O)ccc2[C@H]2CC[C@@]3(C)[C@@H](CC[C@@H]3O)[C@@H]21 |
| Glibenclamide | 5-Chloro-N-[2-(4-{[(E)-(cyclohexylimino)(hydroxy)methyl]sulfamoyl}phenyl) ethyl] - 2-methoxybenzene carboximidic acid | Negative | | O/C(=N/C1CCCCC1)NS(=O)(=O)c1ccc(CC\N=C(/O)c2cc(Cl)ccc2OC)cc1 |
| Glimepiride | 3-Ethyl-4-methyl-N-[2-(4-{[(trans-4-methylcyclohexyl)carbamoyl]sulfamoyl}phenyl)ethyl]- 2-oxo-2,5-dihydro-1H-pyrrole-1-carboxamide | Negative | | CC=1CN(C(=O)NCCc2ccc(cc2)S(=O)(=O)NC(=O)N[C@@H]2CC[C@@H](C)CC2)C(=O)C=1CC |
| GR89696 | (R)-methyl 4-(2-(3,4-dichlorophenyl)acetyl)-3-(pyrrolidin-1-ylmethyl)piperazine-1-carboxylate | Positive | [61,62] | Clc1ccc(cc1Cl)CC(=O)N1CC[N@@](CC1CN1CCCC1)C(=O)OC |

| | | | | |
|---|---|---|---|---|
| Harmin | 7-Methoxy-1-methyl-9H-pyrido[3,4-b]indole | Positive | 63 | COc1ccc2c(c1)[NH]c1c2ccnc1C |
| Hoechst 33342 | 2′-(4-Ethoxyphenyl)-5-(4-methyl-1-piperazinyl)-1H,1′H-2,5′-bibenzimidazole | Efflux transporter substrate | | CN1CCN(CC1)c1cc2nc([NH]c2cc1)c1ccc2[NH]c(nc2c1)c1ccc(OCC)cc1 |
| Hydrochlorothiazide | 6-Chloro-3,4-dihydro-2H-1,2,4-benzothiadiazine7-sulfonamide 1,1-dioxide | Negative | | NS(=O)(=O)c1cc2c(NCNS2(=O)=O)cc1Cl |
| Hydrocortisone | (11β)-11,17,21-Trihydroxypregn-4-ene-3,20-dione | Efflux transporter substrate | | OCC(=O)[C@@]1(O)CC[C@H]2[C@@H]3CCC4=CC(=O)CC[C@]4(C)[C@H]3[C@@H](O)C[C@@]21C |
| Indinavir | (2S)-1-[(2S,4R)-4-Benzyl-2-hydroxy-5-{[(1S,2R)-2-hydroxy-2,3-dihydro-1Hinden-1-yl]amino}-5-oxopentyl]-N-(2-methyl-2-propanyl)-4-(3-pyridinylmethyl)-2-piperazinecarboxamide | Efflux transporter substrate | | O[C@H](CN1CCN(C[C@H]1C(=O)NC(C)(C)C)Cc1cccnc1)C[C@@H](Cc1ccccc1)C(=O)N[C@H]1c2ccccc2C[C@H]1O |
| IPCIT | (1R,2S,3S,5S)-methyl 8-((E)-3-iodoallyl)-3-(4-iodophenyl)-8-azabicyclo[3.2.1]octane-2-carboxylate | Positive | 64–66 | Ic1ccc(cc1)C1CC2CCC(C1C(=O)OC)N2C\C=C\I |
| Itraconazole | 2-sec-Butyl-4-{4-[4-(4-{[(2R,4S)-2-(2,4-dichlorophenyl)- 2-(1H-1,2,4-triazol-1-ylmethyl)-1, 3-dioxolan-4-yl]methoxy}phenyl)-1-piperazinyl]phenyl}- 2,4-dihydro-3H-1,2,4-triazol-3-one | Negative | | CC(CC)N1N=CN(C1=O)c1ccc(cc1)N1CCN(CC1)c1ccc(cc1)OC[C@H]1CO[C@@](Cn2ncnc2)(O1)c1ccc(Cl)cc1Cl |
| Ketoconazole | 1-[4-(4-{[(2R,4S)-2-(2,4-Dichlorophenyl)-2-(1H-imidazol1-ylmethyl)-1,3-dioxolan4-yl]methoxy}phenyl)-1-piperazinyl]ethanone | Negative | | CC(=O)N1CCN(CC1)c1ccc(cc1)OC[C@H]1CO[C@@](Cn2ccnc2)(O1)c1ccc(Cl)cc1Cl |
| Laniquidar | methyl 11-(1-(4-(quinolin-2-ylmethoxy)phenethyl)piperidin-4-ylidene)-6,11-dihydro-5H-benzo[d]imidazo[1,2-a]azepine-3-carboxylate | Efflux transporter substrate | 67 | O=C(OC)c1cnc2n1CCc1ccccc1\C2=C1/CCN(CCc2ccc(OCc3ccc4ccccc4n3)cc2)CC1 |

| | | | | |
|---|---|---|---|---|
| Linagliptin | 8-(3-Amino-1-piperidinyl)-7-(2-butyn-1-yl)-3-methyl-1- [(4-methyl-2-quinazolinyl)methyl]- 3,7-dihydro-1H-purine-2,6-dione | Negative | | NC1CCCN(C1)c1nc2c(C(=O)N(Cc3nc4ccccc4c(C)n3)C(=O)N2C)n1CC#CC |
| Loperamide | 4-[4-(4-Chlorophenyl)-4-hydroxy-1-piperidinyl]- N,Ndimethyl-2,2-diphenylbutanamide | Efflux transporter substrate | 68 | CN(C)C(=O)C(CCN1CCC(O)(CC1)c1ccc(Cl)cc1)(c1ccccc1)c1ccccc1 |
| Lopinavir | (2S)-N-[(2S,4S,5S)-5-{[(2,6-Dimethylphenoxy)acetyl]amino}-4-hydroxy-1,6- diphenyl-2-hexanyl]-3-methyl-2-(2-oxotetrahydro-1(2H)-pyrimidinyl)butanamide | Efflux transporter substrate | | CC(C)[C@@H](C(=O)N[C@@H](Cc1ccccc1)C[C@H](O)[C@H](Cc1ccccc1)NC(=O)COc1c(C)cccc1C)N1CCCNC1=O |
| Loratadine | Ethyl 4-(8-chloro-5,6-dihydro-11Hbenzo[5,6]cyclohepta[1,2-b]pyridin-11-ylidene)-1-piperidinecarboxylate | Negative | | Clc1cc2CCc3cccnc3C(=C3/CCN(CC3)C(=O)OCC)\c2cc1 |
| Losartan | (2-Butyl-4-chloro-1-{[2′-(1H-tetrazol-5-yl)-4-biphenylyl]methyl}- 1H-imidazol-5-yl)methano | Efflux transporter substrate | | OCc1c(Cl)nc(CCCC)n1Cc1ccc(cc1)c1ccccc1c1nnn[NH]1 |
| McN 5652 | (6S,10bR)-6-(4-(methylthio)phenyl)-1,2,3,5,6,10bhexahydropyrrolo[2,1-a]isoquinoline | Positive | 69,70 | CSc1ccc(cc1)[C@@H]1CN2CCCC2c2ccccc21 |
| MDL (Volinanserin) | rac-(R)-(2,3-dimethoxyphenyl)(1-(4-fluorophenethyl)piperidin-4-yl)methanol | Positive | 71,72 | COc1cccc(c1OC)[C@H](O)C1CCN(CCc2ccc(F)cc2)CC1 |
| MDL100151 | 2,3-Dimethoxyphenyl)(1-(4-fluorophenethyl) piperidin-4-yl)methanol | Positive | 73 | COc1cccc(c1OC)C(O)C1CCN(CCc2ccc(F)cc2)CC1 |
| MDL100907 | (R)-(2,3-dimethoxyphenyl)(1-(4-fluorophenethyl) piperidin-4-yl)methanol | Positive | 69 | COc1cccc(c1OC)[C@H](O)C1CCN(CCc2ccc(F)cc2)CC1 |

| | | | | |
|---|---|---|---|---|
| Me@HAPTHI | 4-(2,2-Dioxido-3-phenyl-2,1,3-benzothiadiazol-1(3H)-yl)-1-(methylamino)-2-butanol | Negative | [74] | CNC[C@@H](O)CCN1c2ccccc2N(c2ccccc2)S1(=O)=O |
| Me@IPICIT | Methyl (1R,2S,3S,5S)-3-(4-iodophenyl)-8-[(2E)-3-iodo-2-propen-1-yl]-8-azabicyclo [3.2.1]octane-2-carboxylate | Positive | Visual classification (mouse, unpublished) | Ic1ccc(cc1)C1CC2CCC(C1C(=O)OC)N2C\C=C\I |
| Mesna (Coenzyme M) | 2-mercaptoethane sulfonic acid | Negative | | [O-]S(=O)(=O)CCS |
| Metaraminol | rac-3-((1R,2S)-2-amino-1-hydroxypropyl)phenol | Negative | | O[C@H](c1cc(O)ccc1)[C@H](C)N |
| Methylnaltrexone bromide | (3S,4S,4aS,7aR,12bS)-3-(cyclopropylmethyl)-4a,9-dihydroxy-3-methyl-7-oxo-2,3,4,4a,5,6,7,7a-octahydro-1H-4,12-methanobenzofuro[3,2-e]isoquinolin-3-ium | Negative | | C[N+]1(CC2CC2)CC[C@]23c4c5O[C@H]2C(=O)CC[C@@]3(O)[C@@H]1Cc4ccc5O |
| Metomidate | (R)-methyl 1-(1-phenylethyl)-1H-imidazole-5-carboxylate | Positive | [49,75] | C[C@H](c1ccccc1)n1cncc1C(=O)OC |
| mHED | 3-[1-Hydroxy-2-(methylamino)propyl]phenol | Negative | [76,77] | O[C@H](c1cc(O)ccc1)[C@H](C)N[11CH3] |
| Miconazol | 1-{2-[(2,4-Dichlorobenzyl)oxy]-2-(2,4-dichlorophenyl)ethyl}-1H-imidazole | Negative | | Clc1ccc(C(OCc2ccc(Cl)cc2Cl)Cn2ccnc2)c(Cl)c1 |
| Mitoxantrone | 1,4-dihydroxy-5,8-bis((2-((2-hydroxyethyl)amino)ethyl)amino)anthracene-9,10-dione | Efflux transporter substrate | | OCCNCCNc1ccc(NCCNCCO)c2C(=O)c3c(O)ccc(O)c3C(=O)c21 |

| | | | | |
|---|---|---|---|---|
| MPPF | 4-Fluoro-N-(2-(4-(2-methoxyphenyl)piperazin-1-yl)ethyl)- N-(pyridin-2-yl)benzamide | Positive | 78,79 | Fc1ccc(cc1)C(=O)N(CCN1CCN(CC1)c1ccccc1OC)c1ccccn1 |
| Nadolol | (2R,3S)-5-{2-Hydroxy-3-[(2-methyl-2-propanyl)amino]propoxy}-1,2,3,4-tetrahydro-2, 3-naphthalenediol | Negative | | CC(C)(C)NCC(O)COc1cccc2C[C@@H](O)[C@@H](O)Cc21 |
| N-ButylScopalamine | (1RS,2RS,4SR,5SR,7SR,9R)-9-butyl-7-(((SR)-3-hydroxy-2-phenylpropanoyl)oxy)-9-methyl-3-oxa-9-azatricyclo[3.3.1.02,4]nonan-9-ium | Negative | | OC[C@H](c1ccccc1)C(=O)O[C@@H]1C[C@@H]2[C@H]3O[C@H]3[C@H](C1)[N+]2(C)CCCC |
| Nelfinavir | (3S,4aS,8aS)-2-[(2R,3R)-2-Hydroxy-3-[(3-hydroxy-2-methylbenzoyl)amino]-4-(phenylsulfanyl)butyl]-N-(2-methyl-2-propanyl)decahydro3-isoquinolinecarboxamide | Efflux transporter substrate | | Oc1cccc(c1C)C(=O)N[C@@H](CSc1ccccc1)[C@H](O)CN1C[C@H]2CCCC[C@H]2C[C@H]1C(=O)NC(C)(C)C |
| Neostigmine | 3-[(Dimethylcarbamoyl)oxy]- N,N,N-trimethylanilinium | Negative | | O=C(Oc1cccc(c1)[N+](C)(C)C)N(C)C |
| Nicardipine | 2-[Benzyl(methyl)amino]ethyl methyl 2,6-dimethyl-4-(3-nitrophenyl)-1, 4-dihydro-3,5-pyridinedicarboxylate | Efflux transporter substrate | | CC=1NC(C)=C(C(C=1C(=O)OCCN(C)Cc1ccccc1)c1cccc(c1)[N+]([O-])=O)C(=O)OC |
| Nifene | 3-((2,5-dihydro-1H-pyrrol-2-yl)methoxy)-2-fluoropyridine | Positive | 80,81 | Fc1ncccc1OCC1C=CCN1 |
| N-Methylnaltrinidole | (8aS,14bR)-7-(cyclopropylmethyl)-14-methyl-5,6,7,8,8a,9,14, 14b-octahydro-4,8-methanobenzofuro[2,3-a]pyrido [4,3-b]carbazole-1,8a-diol | Positive | 82 | Cn1c2ccccc2c2C[C@@]3(O)C4Cc5ccc(O)c6O[C@@H](c21)C3(CCN4CC1CC1)c65 |
| NNC precursor | (5S)-5-(1-Benzofuran-7-yl)-8-chloro-2,3,4,5-tetrahydro-1H-3-benzazepin-7-ol | Positive | | Clc1cc2CCNC[C@H](c3cccc4ccoc34)c2cc1O |

| | | | | |
|---|---|---|---|---|
| NNC112 | (S)-5-(benzofuran-7-yl)-8-chloro-3-methyl-2,3,4,5-tetrahydro-1H-benzo[d]azepin-7-ol | Positive | 83 | CN1CCC2=CC(=C(C=C2[C@H](C1)C3=CC=CC4=C3OC=C4)O)Cl |
| Ondansetron | 9-Methyl-3-[(2-methyl-1H-imidazol-1-yl)methyl]- 1,2,3,9-tetrahydro-4H-carbazol-4-one | Efflux transporter substrate | | O=C1C(CCc2c1c1ccccc1n2C)Cn1ccnc1C |
| Parecoxib | N-{[4-(5-Methyl-3-phenyl-1,2-oxazol4-yl)phenyl]sulfonyl}propanamide | Negative | | CCC(=O)NS(=O)(=O)c1ccc(cc1)c1c(C)onc1c1ccccc1 |
| PE2i | (1R,2S,3S,5S)-methyl 8-((E)-3-iodoallyl)-3-(p-tolyl)-8- azabicyclo[3.2.1]octane-2-carboxylate | Positive | 84 | Cc1ccc(cc1)[C@H]1C[C@@H]2CC[C@H]([C@H]1C(=O)OC)N2C\C=C\I |
| Phenylbutazone | 4-Butyl-1,2-diphenyl-3,5-pyrazolidinedione | Negative | | O=C1C(CCCC)C(=O)N(c2ccccc2)N1c1ccccc1 |
| PHNO | (4R,4aR,10bR)-9-hydroxy-4-propyl-3,4,4a,5,6,10b-hexahydro2H-naphtho[1,2-b] [1, 4]oxazin-4-ium | Positive | 85 | Oc1ccc2CC[C@@H]3[C@H](OCCN3CCC)c2c1 |
| Physostigmine | (3aS,8aR)-1,3a,8-trimethyl-1,2,3,3a,8,8a-hexahydropyrrolo[2,3-b]indol-5-yl methylcarbamate | Positive | 86–88 | CNC(=O)Oc1cc2c(cc1)N(C)[C@H]1N(C)CC[C@@]21C |
| PK11195 | N-(sec-butyl)-1-(2-chlorophenyl)-N-methylisoquinoline3-carboxamide | Positive | 89 | CC(CC)N(C)C(=O)c1cc2ccccc2c(n1)c1ccccc1Cl |
| PMP | piperidin-4-yl propionate | Positive | 90,91 | O=C(OC1CCNCC1)CC |

| | | | | |
|---|---|---|---|---|
| Polymyxin B2 | rac-N-((R)-4-amino-1-(((2R,3S)-1-(((R)-4-amino-1-oxo-1-(((3R,6R,9R,12R,15S,18R,21R)-6,9,18-tris(2-aminoethyl)-15-benzyl-3-((S)-1-hydroxyethyl)-12-isobutyl-2,5,8,11,14,17,20-heptaoxo-1,4,7,10,13,16,19-heptaazacyclotricosan-21-yl)amino)butan-2-yl)amino)-3-hydroxy-1-oxobutan-2-yl)amino)-1-oxobutan-2-yl)-6-methylheptanamide | Negative | | CC(C)CCCCC(=O)N[C@@H](CCN)C(=O)N[C@@H]([C@@H](C)O)C(=O)N[C@@H](CCN)C(=O)N[C@H]1CCNC(=O)[C@@H](NC(=O)[C@H](CCN)NC(=O)[C@H](CCN)NC(=O)[C@H](CC(C)C)NC(=O)[C@@H](Cc2ccccc2)NC(=O)[C@@H](NC1=O)CCN)[C@@H](C)O |
| Quinacrine | N4-(6-Chloro-2-methoxy-9-acridinyl)-N1,N1-diethyl-1,4-pentanediamine | Efflux transporter substrate | | CCN(CC)CCCC(C)Nc1c2cc(OC)ccc2nc2cc(Cl)ccc12 |
| Quinidine | (9S)-6′-Methoxycinchonan-9-ol | Efflux transporter substrate | | COc1cc2c(cc1)nccc2[C@H](O)C1CC2CCN1CC2C=C |
| Raclopride | (S)-3,5-dichloro-N-((1-ethylpyrrolidin-2-yl)methyl)-2- hydroxy-6-methoxybenzamide | Positive | [92,93] | CCN1CCC[C@H]1CNC(=O)c1c(O)c(Cl)cc(Cl)c1OC |
| Ranitidine | (E)-N-{2-[({5-[(Dimethylamino)methyl]-2-furyl}methyl)sulfanyl]ethyl}- N′- methyl-2-nitro-1,1-ethenediamine | Efflux transporter substrate | | [O-][N+](=O)\C=C(/NC)NCCSCc1ccc(CN(C)C)o1 |
| Repaglinid | 2-Ethoxy-4-[2-({(1S)-3-methyl-1-[2- (1-piperidinyl)phenyl]butyl}amino)- 2-oxoethyl]benzoic acid | Negative | | O=C(O)c1ccc(cc1OCC)CC(=O)N[C@@H](CC(C)C)c1ccccc1N1CCCCC1 |
| Rhodamine 123 | 6-Amino-9-[2-(methoxycarbonyl)phenyl]-3H-xanthen-3-iminium | Efflux transporter substrate | | O=C(OC)c1ccccc1C=1c2ccc(N)cc2OC2=CC(=[NH2+])C=CC=12 |
| Ritalin | rac-methyl (R)-2-phenyl-2-((R)-piperidin-2-yl)acetate | Positive | [94] | O=C(OC)[C@@H]([C@H]1CCCCN1)c1ccccc1 |

| | | | | |
|---|---|---|---|---|
| Ritonavir | (1E,2S)-N-[(2S,4S,5S)-4-Hydroxy-5-{(E)-[hydroxy(1,3-thiazol-5-ylmethoxy) methylene]amino}-1,6-diphenyl-2-hexanyl]-2-[(E)- (hydroxy{[(2-isopropyl-1,3-thiazol-4-yl)methyl](methyl)amino}methylene)amino]- 3- methylbutanimidic acid | Efflux transporter substrate | | CC(C)c1nc(CN(C)C(/O)=N\[C@H](C(/O)=N\[C@@H](Cc2ccccc2)C[C@H](O)[C@H](Cc2ccccc2)/N=C(\O)OCc2cncs2)C(C)C)cs1 |
| Ro11-2933/Acetanilide | N-[2-(3,4-Dimethoxyphenyl)ethyl]- N-methyl-3-[2-(2-naphthyl)- 1,3-dithian-2-yl]-1-propanamine | Efflux transporter substrate | | COc1ccc(cc1OC)CCN(C)CCCC1(SCCCS1)c1ccc2ccccc2c1 |
| Rofecoxib | 4-[4-(Methylsulfonyl)phenyl]-3-phenyl2(5H)-furanone | Negative | | CS(=O)(=O)c1ccc(cc1)C=1COC(=O)C=1c1ccccc1 |
| Rolipram | 4-(3-(Cyclopentyloxy)-4-methoxyphenyl)pyrrolidin-2-one | Positive | 95–97 | COc1ccc(cc1OC1CCCC1)C1CC(=O)NC1 |
| Roxithromycin | (3R,4S,5S,6R,7R,9R,10E,11S,12R,13S,14R)- 6-{[(2S,3R,4S,6R)-4-(Dimethylamino)-3-hydroxy-6-methyltetrahydro-2H-pyran2-yl]oxy}-14-ethyl-7,12,13-trihydroxy-4-{[(2R,4R,5S,6S)-5-hydroxy-4-methoxy-4,6-dimeth yltetrahydro-2H-pyran-2-yl]oxy}-10-{[(2-methoxyethoxy)methoxy]imino}-,5,7,9,11,13-hexamethyloxacyclotetradecan-2-one(3R)-3-Amino-1-[3-(trifluoromethyl)-5,6 | Negative | | CN(C)[C@H]1C[C@@H](C)O[C@@H](O[C@@H]2[C@@H](C)[C@H](O[C@H]3C[C@@](C)(OC)[C@@H](O)[C@H](C)O3)[C@@H](C)C(=O)O[C@H](CC)[C@@](C)(O)[C@H](O)[C@@H](C)\C(=N\OCOCCOC)[C@H](C)C[C@@]2(C)O)[C@@H]1O |
| SB207145 | (1-methylpiperidin-4-yl)methyl 8-amino-7-chloro-2,3-dihydrobenzo[b][1,4]dioxine-5-carboxylate | Positive | 98,99 | CN1CCC(CC1)COC(=O)c1cc(Cl)c(N)c2OCCOc21 |
| SCH-23388 hydrochloride | (S)-8-Chloro-3-methyl-5-phenyl-2,3,4,5-tetrahydro1H-benzo[d]azepin-7-ol | Positive | | Clc1cc2CCN(C)C[C@H](c2cc1O)c1ccccc1 |
| SCH-23390 hydrochloride | (R)-8-chloro-3-methyl-5-phenyl-2,3,4,5-tetrahydro1H-benzo[d]azepin-7-ol | Positive | 100 | Clc1cc2CCN(C)C[C@@H](c2cc1O)c1ccccc1 |

| | | | | |
|---|---|---|---|---|
| Scopalamine | (1R,5S)-9-methyl-3-oxa-9-azatricyclo[3.3.1.02,4]nonan-7-yl (2S)-3-hydroxy-2-phenylpropanoate | Positive | 101 | OC[C@H](c1ccccc1)C(=O)OC1C[C@H]2N(C)[C@@H](C1)C1OC21 |
| Setoperone | 6-(2-(4-(4-Fluorobenzoyl)piperidin-1-yl)ethyl)-7- methyl-2H-thiazolo[3,2-a]pyrimidin-5(3H)-one | Positive | 102,103 | Fc1ccc(cc1)C(=O)C1CCN(CC1)CCC1=C(C)N=C2SCCN2C1=O |
| Sitagliptin | (3R)-3-Amino-1-[3-(trifluoromethyl)-5,6-dihydro[1,2,4]triazolo[4,3-a]pyrazin-7(8H)-yl]-4-(2,4,5-trifluorophenyl)-1-butanone | Negative | | C1CN2C(=NN=C2C(F)(F)F)CN1C(=O)C[C@@H](CC3=CC(=C(C=C3F)F)F)N |
| SNAP7941 | methyl 3-((3-(4-(3-acetamidophenyl)piperidin-1-yl)propyl)carbamoyl)-4-(3,4-difluorophenyl)-6-(methoxymethyl)-2-oxo-1,2,3,4-tetrahydropyrimidine-5-carboxylate | Efflux transporter substrate | 104,105 | O=C(OC)C1=C(COC)NC(=O)N(C1c1ccc(F)c(F)c1)C(=O)NCCCN1CCC(CC1)c1cccc(NC(C)=O)c1 |
| Sotalol | N-{4-[1-Hydroxy-2-(isopropylamino)ethyl]phenyl}methanesulfonamide | Negative | | O=S(C)(=O)Nc1ccc(cc1)C(O)CNC(C)C |
| Tariquidar | N-[2-({4-[2-(6,7-Dimethoxy-3,4-dihydro-2(1H)-isoquinolinyl)ethyl]phenyl} carbamoyl)-4,5-dimethoxyphenyl]-3-quinolinecarboxamide | Efflux transporter substrate | | COc1cc(c(NC(=O)c2cc3ccccc3nc2)cc1OC)C(=O)Nc1ccc(cc1)CCN1Cc2cc(OC)c(cc2CC1)OC |
| Teicoplanin | (1S,2R,19R,22R,34S,37R,40R,52S)-2-[(2R,3R,4R,5S,6R)-3-acetamido-4,5-dihydroxy-6-(hydroxymethyl)oxan-2-yl]oxy-22-amino-5,15-dichloro-64-[(2S,3R,4R,5S,6R)-3-(decanoylamino)-4,5-dihydroxy-6-(hydroxymethyl)oxan-2-yl]oxy-26,31,44,49-tetrahydroxy-21,35,38,54,56,59-hexaoxo-47-[(3S,4S,5S,6R)-3,4,5-trihydroxy-6-(hydroxymethyl)oxan-2-yl]oxy-7,13,28-trioxa-20,36,39,53,55,58-hexazaundecacyclo[38.14.2.23,6.214,17.219,34.18,12.123,27.129,33.141,45.010,37.046,51]hexahexaconta-3,5,8,10,12(64),14,16,23(61),24,26,29(60),30,32,41(57),42,44,46(51),47,49,62,65-henicosaene-52-carboxylic acid | Negative | | CCCCCCCCCC(=O)N[C@@H]1[C@H]([C@@H]([C@H](O[C@H]1OC2=C3C=C4C=C2OC5=C(C=C(C=C5)[C@H]([C@H]6C(=O)N[C@@H](C7=C(C(=CC(=C7)O)OC8[C@H]([C@H]([C@@H]([C@H](O8)CO)O)O)O)C9=C(C=CC(=C9)[C@H](C(=O)N6)NC(=O)[C@@H]4NC(=O)[C@@H]1C2=CC(=CC(=C2)OC2=C(C=CC(=C2)[C@H](C(=O)N[C@H](CC2=CC(=C(O3)C=C2)Cl)C(=O)N1)N)O)O)O)C(=O)O)O[C@H]1[C@@H]([C@H]([C@@H]([C@H](O1)CO)O)O)NC(=O)C)Cl)CO)O)O |

| | | | | |
|---|---|---|---|---|
| Teniposide | (5S,5aR,8aR,9R)-9-(4-Hydroxy-3,5-dimethoxyphenyl)-8-oxo-5,5a,6,8,8a,9-hexahydrofuro[3′,4′:6,7]naphtho[2,3-d][1,3]dioxol-5-yl 4,6-O-(2-thienylmethylene)-β-D-glucopyranoside | Efflux transporter substrate | | COc1cc(cc(OC)c1O)[C@@H]1c2cc3OCOc3cc2[C@@H](O[C@@H]2O[C@@H]3COC(O[C@H]3[C@H](O)[C@H]2O)c2cccs2)[C@H]2COC(=O)[C@H]12 |
| Terfenadine | 4-{4-[Hydroxy(diphenyl)methyl]-1-piperidinyl}-1-[4-(2-methyl-2-propanyl)phenyl]-1-butanol | Negative | | CC(C)(C)c1ccc(cc1)C(O)CCCN1CCC(CC1)C(O)(c1ccccc1)c1ccccc1 |
| TMSX | (E)-1,3,5-trimethyl-6-(3,4,5-trimethoxystyryl)-1,4a,5,7a-tetrahydro-2H-pyrrolo[2,3-d]pyrimidine-2,4(3H)-dione | Positive | 106 | COc1cc(cc(OC)c1OC)/C=C/C1=NC2C(C(=O)N(C)C(=O)N2C)C1C |
| Tolcapone | (3,4-dihydroxy-5-nitrophenyl)(p-tolyl)methanone | Negative | | Oc1cc(cc([N+]([O-])=O)c1O)C(=O)c1ccc(C)cc1 |
| Triamcinolone | (11β,16α)-9-Fluoro-11,16,17,21-tetrahydroxypregna-1,4-diene-3,20-dione | Efflux transporter substrate | | OCC(=O)[C@@]1(O)[C@@H](O)C[C@H]2[C@@H]3CCC4=CC(=O)C=C[C@]4(C)C3(F)[C@H](O)C[C@@]21C |
| Verapamil | 2-(3,4-Dimethoxyphenyl)-5-{[2-(3,4-dimethoxyphenyl)ethyl](methyl)amino}- 2-isopropylpentanenitrile hydrochloride | Efflux transporter substrate | 107 | COc1cc(ccc1OC)[C@@](C#N)(CCCN(C)CCc1ccc(OC)c(OC)c1)C(C)C |
| Vinblastine | Dimethyl (2β,3β,4β,5α,12β,19α)-15-[(5S,9S)-5-ethyl-5-hydroxy-9-(methoxycarbonyl)-1,4,5,6,7,8,9,10-octahydro-2 H-3,7-methanoazacycloundecino [5,4-b]indol- 9-yl]-3-hydroxy-16-methoxy-1-methyl-6,7-didehydroaspidospermidine3,4-dicarboxylate | Efflux transporter substrate | | O=C(OC)[C@@]1(C[C@@H]2C[C@@](O)(CC)CN(CCc3c4ccccc4[NH]c31)C2)c1cc2c(cc1OC)N(C)[C@@H]1[C@]22CCN3CC=C[C@](CC)([C@H]32)[C@@H](OC(C)=O)[C@]1(O)C(=O)OC |
| Vincristine | (3aR,3a1R,4R,5S,5aR,10bR)-Methyl 4-acetoxy-3a-ethyl-9- ((5S,7S,9S)-5-ethyl-5-hydroxy-9-(methoxycarbonyl)-2,4,5,6,7,8,9,10-octahydro-1 H-3,7-methano[1]azacycloundecino[5,4- b]indol-9-yl)-6-formyl-5-hydroxy-8-methoxy3a,3a1,4,5,5a,6,11,12-octahydro-1 H-indolizino[8,1-cd]carbazole-5-carboxylate | Efflux transporter substrate | | O=C(OC)[C@@]1(C[C@H]2C[C@@](O)(CC)C[N@@](CCc3c4ccccc4[NH]c31)C2)c1cc2c(cc1OC)N(C=O)[C@@H]1[C@]22CCN3CC=C[C@](CC)([C@H]32)[C@@H](OC(C)=O)[C@]1(O)C(=O)OC |

| | | | | |
|---|---|---|---|---|
| WAY-100635 | N-(2-(4-(2-methoxyphenyl)piperazin-1-yl)ethyl)-N-(pyridin-2-yl) | Positive | 108 | COc1ccccc1N1CCN(CCN(c2ccccn2)C(=O)C2CCCCC2)CC1 |

**Table S2.** Characteristics of physico-chemial parameters stratified by CNS positivity. *P* values are exploratory.

| Feature | Entire data | CNS negative | CNS positive | Efflux | p value (CNS negative vs. positive) | p value (CNS negative vs. efflux) | p value (CNS positive vs. efflux) | All groups p value < 0.05 |
|---|---|---|---|---|---|---|---|---|
| Number of compounds, n (%) | 154 (100.00%) | 42 (27.27%) | 68 (44.16%) | 44 (28.57%) | NA | NA | NA | |
| MW (g/mol) | 434.6 (220.07) | 473.29 (311.13) | 346.1 (90.36) | 534.44 (202.42) | 0.0251 | 0.0073 | < 0.0001 | * |
| Freely Rotable Bonds (ACD) | 6.41 (4.37) | 7.5 (6.0) | 4.54 (2.12) | 8.2 (3.93) | 0.0055 | 0.0822 | < 0.0001 | |
| PSA (ACD) | 90.36 (83.62) | 124.28 (125.42) | 48.42 (17.08) | 121.84 (63.94) | < 0.0001 | 0.1338 | < 0.0001 | |
| HBD (ACD) | 2.12 (3.49) | 3.57 (5.76) | 0.67 (0.78) | 2.93 (1.99) | < 0.0001 | 0.1621 | < 0.0001 | |
| HBA (ACD) | 6.58 (5.38) | 8.36 (7.74) | 4.04 (1.44) | 8.75 (4.79) | < 0.0001 | 0.0539 | < 0.0001 | |
| logP (ACD) | 2.89 (2.26) | 2.35 (3.03) | 2.98 (1.46) | 3.24 (2.34) | 0.374 | 0.2087 | 0.6421 | |
| logD (pH7.4) (ACD) | 1.6 (2.52) | 0.5 (3.88) | 2.02 (1.35) | 2.02 (1.84) | 0.0153 | 0.0422 | 0.9115 | |
| HPLC logP-HBA (ACD) | -4.01 (5.36) | -5.61 (7.16) | -1.18 (2.4) | -6.04 (4.73) | < 0.0001 | 0.4718 | < 0.0001 | |
| HBA+HBD (ACD) | 8.69 (8.5) | 11.93 (13.17) | 4.69 (1.63) | 11.68 (6.16) | < 0.0001 | 0.0572 | < 0.0001 | |
| logP-HBA (ACD) | -3.7 (6.41) | -6.0 (9.04) | -1.08 (2.51) | -5.51 (6.04) | < 0.0001 | 0.7591 | < 0.0001 | |
| logD (pH 7.4)-HBA (ACD) | -5.05 (6.93) | -7.94 (10.38) | -2.08 (2.22) | -6.73 (5.72) | < 0.0001 | 0.8638 | < 0.0001 | |
| HPLC logP(pH 7.4) | 2.53 (1.59) | 1.8 (2.09) | 2.88 (1.23) | 2.71 (1.27) | 0.0416 | 0.1051 | 0.5596 | |
| IAM Km (linear) | 137.09 (211.71) | 114.2 (226.82) | 100.06 (65.68) | 198.35 (285.89) | 0.0113 | 0.0331 | 0.7542 | |
| IAM Pm (linear) | 0.31 (0.44) | 0.25 (0.51) | 0.28 (0.18) | 0.39 (0.57) | 0.0009 | 0.0672 | 0.2054 | |
| HSA | 70.07 (20.4) | 69.41 (24.32) | 69.25 (15.59) | 71.55 (21.97) | 0.0871 | 0.7075 | 0.0815 | |
| LogK | 0.45 (0.44) | 0.44 (0.53) | 0.43 (0.33) | 0.5 (0.48) | 0.1606 | 0.8181 | 0.1568 | |
| tPSA | 85.31 (83.59) | 118.43 (126.85) | 45.07 (17.86) | 115.88 (64.19) | < 0.0001 | 0.0998 | < 0.0001 | |
| logP | 3.01 (2.01) | 3.24 (2.45) | 3.05 (1.32) | 2.76 (2.54) | 0.6968 | 0.412 | 0.5199 | |
| ClogP | 3.02 (2.37) | 2.37 (3.01) | 3.19 (1.38) | 3.35 (2.78) | 0.2669 | 0.2527 | 0.7119 | |
| pKa (prediction) | 6.19 (4.95) | 5.54 (5.02) | 5.57 (4.03) | 7.69 (5.75) | 0.9864 | 0.0914 | 0.0272 | |
| 3D PSA | 110.3 (77.26) | 146.19 (93.24) | 62.98 (34.46) | 149.55 (67.81) | < 0.0001 | 0.2884 | < 0.0001 | |
| CNS MPO score | 4.09 (1.26) | 3.91 (1.2) | 4.68 (0.91) | 3.34 (1.32) | 0.0011 | 0.0448 | < 0.0001 | * |
| BBB score | 3.87 (1.34) | 3.31 (1.31) | 4.88 (0.34) | 2.83 (1.23) | < 0.0001 | 0.0948 | < 0.0001 | |
| CNS MPO PET score | 2.95 (1.38) | 2.79 (1.26) | 3.65 (1.29) | 2.08 (1.02) | 0.0028 | 0.0164 | < 0.0001 | * |

**Metrics**

Area under the receiver operating characteristic curve (AUC):

AUC was calculated via scikit-learn version 1.1.0[15].

Accuracy (ACC):

$$ACC = \frac{\mathrm{TP} + \mathrm{TN}}{\mathrm{TP} + \mathrm{TN} + \mathrm{FP} + \mathrm{FN}}$$

Sensitivity (SNS):

$$SNS = \frac{\mathrm{TP}}{\mathrm{TP} + \mathrm{FN}}$$

Positive predictive value (PPV):

$$PPV = \frac{\mathrm{TP}}{\mathrm{TP} + \mathrm{FP}}$$

Specificity (SPC):

$$SPC = \frac{\mathrm{TN}}{\mathrm{TN} + \mathrm{FP}}$$

Negative predictive value (NPV):

$$NPV = \frac{\mathrm{TN}}{\mathrm{TN} + \mathrm{FN}}$$

**Machine learning parameters and hyperparameter grids**

Parameter options for hyperparameter search are separated via a comma.

<u>Preprocessing</u>

max_missing_ratio = 0.3

number_of_selected_features = sqrtn

imputation_method = knn

<u>Training</u>

number_of_folds = 100

test_set_ratio = 0.2

randomizedsearchcv_cv = 5

randomizedsearchcv_n_iter = 10

<u>EBM hyperparameters</u>

feature_names = None

feature_types = None

max_bins = 256

max_interaction_bins = 64

binning = quantile

mains = all

interactions = 5

outer_bags = 8, 16

inner_bags = 0, 8

learning_rate = 0.01, 0.001, 0.0001

validation_size = 0.15

early_stopping_rounds = 50

early_stopping_tolerance = 0.0001

max_rounds = 10

min_samples_leaf = 2, 4

max_leaves = 3

n_jobs = -2

random_state = 0

<u>KNN hyperparameters</u>

weights = distance

algorithm = auto

leaf_size = 30

p = 1, 2, 3, 4, 5

metric = minkowski

metric_params = None

n_jobs = -1

<u>DT hyperparameters</u>

criterion = gini

splitter = best, random

max_depth = 1, 2, 3, 4, 5, 7, 10, 15

min_samples_split = 2, 4, 6

min_samples_leaf = 1, 3, 5

min_weight_fraction_leaf = 0.0

max_features = auto, sqrt, log2

random_state = 0

max_leaf_nodes = None

min_impurity_decrease = 0.0

class_weight = None

ccp_alpha = 0.0

RF hyperparameters

n_estimators = 100

criterion = entropy

max_depth = 2, 4, 5, 10, 15

min_samples_split = 2, 4, 8

min_samples_leaf = 1, 3, 5, 7

min_weight_fraction_leaf = 0.0

max_features = auto, sqrt, log2

max_leaf_nodes = None

min_impurity_decrease = 0.0

bootstrap = True

oob_score = False

n_jobs = -1

random_state = 0

verbose = 0

warm_start = False

class_weight = None

ccp_alpha = 0.0

max_samples = None

XGB hyperparameters

objective = binary:logistic

use_label_encoder = False

base_score = None

booster = None

callbacks = None

colsample_bylevel = None

colsample_bynode = None

colsample_bytree = 0.5, 0.7, 1

early_stopping_rounds = None

enable_categorical = False

eval_metric = None

gamma = 0, 0.2

gpu_id = None

grow_policy = None

importance_type = None

interaction_constraints = None

learning_rate = 0.2, 0.3

max_bin = None

max_cat_to_onehot = None

max_delta_step = None

max_depth = 2, 4, 6, 8

max_leaves = None

min_child_weight = 1, 3

missing = nan

monotone_constraints = None

n_estimators = 100

n_jobs = -1

num_parallel_tree = None

predictor = None

random_state = 0

reg_alpha = None

reg_lambda = None

sampling_method = None

scale_pos_weight = None

subsample = None

tree_method = None

validate_parameters = None

verbosity = None

SVM hyperparameters

C = 0.1, 1.0, 10

kernel = rbf, poly, sigmoid, linear

degree = 2, 3, 4

gamma = scale, auto

coef0 = 0.0

shrinking = True

probability = True

tol = 0.001

cache_size = 200

class_weight = None

verbose = False

max_iter = -1

decision_function_shape = ovr

break_ties = False

random_state = 0

LGR hyperparameters

penalty = l2

dual = False

tol = 0.0001, 0.001, 0.00001

C = 0.1, 1, 10

fit_intercept = True

intercept_scaling = 1

class_weight = None

random_state = None

solver = lbfgs, liblinear

max_iter = 100

multi_class = auto

verbose = 0

warm_start = False

n_jobs = None

l1_ratio = None

Surrogate DT hyperparameters

criterion = gini

splitter = best

max_depth = 3

min_samples_split = 2

min_samples_leaf = 3

min_weight_fraction_leaf = 0.0

max_features = None

random_state = 0

max_leaf_nodes = None

min_impurity_decrease = 0.0

class_weight = None

ccp_alpha = 0.0

**References**


1. Philippe, C. Preparation and First Preclinical Evaluation of [18F]FE@SNAP: A Potential PET Tracer for the Melanin Concentrating Hormone Receptor 1 (MCHR1). *Scientia Pharmaceutica* **81**, 625–639 (2013).
2. Philippe, C. *et al.* [18F]FE@SNAP—a specific PET tracer for melanin-concentrating hormone receptor 1 imaging? *EJNMMI Research* **6**, (2016).
3. Balber, T. *et al.* Preclinical *In Vitro* and *In Vivo* Evaluation of [ $^{18}$ F]FE@SUPPY for Cancer PET Imaging: Limitations of a Xenograft Model for Colorectal Cancer. *Contrast Media & Molecular Imaging* **2018**, 1–9 (2018).
4. Haeusler, D. *et al.* [18F]FE@SUPPY: a suitable PET tracer for the adenosine A3 receptor? An in vivo study in rodents. *Eur. J. Nucl. Med. Mol. Imaging* **42**, 741–749 (2015).
5. Leung, K. 3-β-(4-Iodophenyl)tropane-2-β-carboxylic acid 2-[18F]fluoroethyl ester. in *Molecular Imaging and Contrast Agent Database (MICAD)* (National Center for Biotechnology Information (US), Bethesda (MD), 2004).
6. Tsukada, H. *et al.* Ketamine alters the availability of striatal dopamine transporter as measured by [(11)C]beta-CFT and [(11)C]beta-CIT-FE in the monkey brain. *Synapse* **42**, 273–280 (2001).
7. Rabinovici, G. D. *et al.* 11C-PIB PET imaging in Alzheimer disease and frontotemporal lobar degeneration. *Neurology* **68**, 1205–1212 (2007).
8. Zhang, S. *et al.* 11C-PIB-PET for the early diagnosis of Alzheimer's disease dementia and other dementias in people with mild cognitive impairment (MCI). *Cochrane Database Syst Rev* **2014**, CD010386 (2014).
9. Hintermann, S. *et al.* ABP688, a novel selective and high affinity ligand for the labeling of mGlu5 receptors: Identification, in vitro pharmacology, pharmacokinetic and biodistribution studies. *Bioorganic & Medicinal Chemistry* **15**, 903–914 (2007).
10. Ametamey, S. M. *et al.* Human PET studies of metabotropic glutamate receptor subtype 5 with 11C-ABP688. *J. Nucl. Med.* **48**, 247–252 (2007).
11. Tan, P. Z. *et al.* Characterization of radioactive metabolites of 5-HT2A receptor PET ligand [18F]altanserin in human and rodent. *Nucl Med Biol* **26**, 601–608 (1999).
12. Pinborg, L. H. *et al.* [18F]altanserin binding to human 5HT2A receptors is unaltered after citalopram and pindolol challenge. *J Cereb Blood Flow Metab* **24**, 1037–1045 (2004).

13. Maeda, J. *et al.* Visualization of alpha5 subunit of GABAA/benzodiazepine receptor by 11C Ro15-4513 using positron emission tomography. *Synapse* **47**, 200–208 (2003).

14. Huang, T. *et al.* The influence of residual nor-β-CFT in 11C CFT injection on the Parkinson disease diagnosis: a 11C CFT PET study. *Clin Nucl Med* **37**, 743–747 (2012).

15. Baldwin, R. M. *et al.* Regional brain uptake and pharmacokinetics of [123I]N-omega-fluoroalkyl-2 beta-carboxy-3 beta-(4-iodophenyl)nortropane esters in baboons. *Nucl Med Biol* **22**, 211–219 (1995).

16. Schönbächler, R. D. *et al.* PET imaging of dopamine transporters in the human brain using [(11)C]-beta-CPPIT, a cocaine derivative lacking the 2 beta-ester function. *Nucl Med Biol* **29**, 19–27 (2002).

17. Kuhl, D. E. *et al.* In vivo butyrylcholinesterase activity is not increased in Alzheimer's disease synapses. *Ann Neurol* **59**, 13–20 (2006).

18. Doze, P. *et al.* Validation of S-1'-[18F]fluorocarazolol for in vivo imaging and quantification of cerebral beta-adrenoceptors. *Eur J Pharmacol* **353**, 215–226 (1998).

19. van Waarde, A. *et al.* Imaging beta-adrenoceptors in the human brain with (S)-1'-[18F]fluorocarazolol. *J Nucl Med* **38**, 934–939 (1997).

20. Endres, C. J., Bencherif, B., Hilton, J., Madar, I. & Frost, J. J. Quantification of brain mu-opioid receptors with [11C]carfentanil: reference-tissue methods. *Nucl. Med. Biol.* **30**, 177–186 (2003).

21. Garcia Guerra, S., Spadoni, A., Mitchell, J. & Strigo, I. A. Pain-related opioidergic and dopaminergic neurotransmission: Dual meta-Analyses of PET radioligand studies. *Brain Res* **1805**, 148268 (2023).

22. Antonini, A. *et al.* The status of dopamine nerve terminals in Parkinson's disease and essential tremor: a PET study with the tracer [11-C]FE-CIT. *Neurol Sci* **22**, 47–48 (2001).

23. Lundkvist, C., Halldin, C., Ginovart, N., Swahn, C. G. & Farde, L. [18F] beta-CIT-FP is superior to [11C] beta-CIT-FP for quantitation of the dopamine transporter. *Nucl Med Biol* **24**, 621–627 (1997).

24. Jakobson Mo, S. *et al.* Dopamine transporter imaging with [18F]FE-PE2I PET and [123I]FP-CIT SPECT-a clinical comparison. *EJNMMI Res* **8**, 100 (2018).

25. Bauer, A. *et al.* In vivo imaging of adenosine A1 receptors in the human brain with [18F]CPFPX and positron emission tomography. *Neuroimage* **19**, 1760–1769 (2003).

26. Elmenhorst, D., Meyer, P. T., Matusch, A., Winz, O. H. & Bauer, A. Caffeine occupancy of human cerebral A1 adenosine receptors: in vivo quantification with 18F-CPFPX and PET. *J Nucl Med* **53**, 1723–1729 (2012).

27. Meyer, P. T. *et al.* Quantification of Cerebral A1 Adenosine Receptors in Humans using [18F]CPFPX and PET: *Journal of Cerebral Blood Flow & Metabolism* 323–333 (2004) doi:10.1097/01.WCB.0000110531.48786.9D.

28. Leung, K. N-Acetyl-N-(2-[18F]fluoroethoxybenzyl)-2-phenoxy-5-pyridinamine. in *Molecular Imaging and Contrast Agent Database (MICAD)* (National Center for Biotechnology Information (US), Bethesda (MD), 2004).

29. Leung, K. N-(5-Fluoro-2-phenoxyphenyl)-N-(2-[131I]iodo-5-methoxybenzyl)acetamide. in *Molecular Imaging and Contrast Agent Database (MICAD)* (National Center for Biotechnology Information (US), Bethesda (MD), 2004).

30. Ginovart, N., Wilson, A. A., Meyer, J. H., Hussey, D. & Houle, S. [11C]-DASB, a tool for in vivo measurement of SSRI-induced occupancy of the serotonin transporter: PET characterization and evaluation in cats. *Synapse* **47**, 123–133 (2003).

31. Gryglewski, G. *et al.* Simple and rapid quantification of serotonin transporter binding using [ 11 C]DASB bolus plus constant infusion. *NeuroImage* **149**, 23–32 (2017).

32. Leung, K. [6-O-methyl-11C]Diprenorphine. in *Molecular Imaging and Contrast Agent Database (MICAD)* (National Center for Biotechnology Information (US), Bethesda (MD), 2004).

33. Levinstein, M. R. *et al.* 6-O-(2-[18F]Fluoroethyl)-6-O-Desmethyl-Diprenorphine ([18F]FE-DPN) Preferentially Binds to Mu Opioid Receptors In Vivo. *Mol Imaging Biol* **25**, 384–390 (2023).

34. Olson, K. M. *et al.* Delta Opioid Receptor-Mediated Antidepressant-Like Effects of Diprenorphine in Mice. *J Pharmacol Exp Ther* **384**, 343–352 (2023).

35. Bauer, M. *et al.* Whole-Body Distribution and Radiation Dosimetry of 11C-Elacridar and 11C-Tariquidar in Humans. *J Nucl Med* **57**, 1265–1268 (2016).

36. Dörner, B. *et al.* Radiosynthesis and in vivo evaluation of 1-[18F]fluoroelacridar as a positron emission tomography tracer for P-glycoprotein and breast cancer resistance protein. *Bioorg. Med. Chem.* **19**, 2190–2198 (2011).

37. Langer, O. *et al.* Carbon-11 epidepride: a suitable radioligand for PET investigation of striatal and extrastriatal dopamine D2 receptors. *Nucl Med Biol* **26**, 509–518 (1999).

38. Almeida, P. *et al.* Absolute quantitation of iodine-123 epidepride kinetics using single-photon emission tomography: comparison with carbon-11 epidepride and positron emission tomography. *Eur J Nucl Med* **26**, 1580–1588 (1999).

39. Tournier, N. *et al.* Complete inhibition of ABCB1 and ABCG2 at the blood-brain barrier by co-infusion of erlotinib and tariquidar to improve brain delivery of the model ABCB1/ABCG2 substrate [11C]erlotinib. *J Cereb Blood Flow Metab* **41**, 1634–1646 (2021).

40. Henriksen, G. *et al.* Syntheses, biological evaluation, and molecular modeling of 18F-labeled 4-anilidopiperidines as mu-opioid receptor imaging agents. *J Med Chem* **48**, 7720–7732 (2005).

41. Justesen, T. E. H., Borghammer, P., Aanerud, J., Hovind, P. & Marner, L. Sertraline treatment influences [18F]FE-PE2I PET imaging for Parkinsonism. *EJNMMI Res* **13**, 46 (2023).

42. Kerstens, V. S. *et al.* [18F]FE-PE2I DAT correlates with Parkinson's disease duration, stage, and rigidity/bradykinesia scores: a PET radioligand validation study. *EJNMMI Res* **13**, 29 (2023).

43. The MICAD Research Team. 4-Bromo-1-(3-[18F]fluoropropyl)-2-nitroimidazole. in *Molecular Imaging and Contrast Agent Database (MICAD)* (National Center for Biotechnology Information (US), Bethesda (MD), 2004).

44. Aalto, S. *et al.* The effects of d-amphetamine on extrastriatal dopamine D2/D3 receptors: a randomized, double-blind, placebo-controlled PET study with [11C]FLB 457 in healthy subjects. *Eur J Nucl Med Mol Imaging* **36**, 475–483 (2009).

45. Narendran, R., Himes, M. & Mason, N. S. Reproducibility of post-amphetamine [11C]FLB 457 binding to cortical D2/3 receptors. *PLoS One* **8**, e76905 (2013).

46. Klumpers, U. M. H. *et al.* Parametric [11C]flumazenil images. *Nucl Med Commun* **33**, 422–430 (2012).

47. Richardson, M. P., Koepp, M. J., Brooks, D. J. & Duncan, J. S. 11C-flumazenil PET in neocortical epilepsy. *Neurology* **51**, 485–492 (1998).

48. Lopes Alves, I. *et al.* Pharmacokinetic modeling of [11C]flumazenil kinetics in the rat brain. *EJNMMI Res* **7**, 17 (2017).

49. Mitterhauser, M. *et al.* In vivo and in vitro evaluation of [18F]FETO with respect to the adrenocortical and GABAergic system in rats. *Eur J Nucl Med Mol Imaging* **30**, 1398–1401 (2003).

50. Wright, E. A. *et al.* Absolute Cerebral Blood Flow Infarction Threshold for 3-Hour Ischemia Time Determined with CT Perfusion and 18F-FFMZ-PET Imaging in a Porcine Model of Cerebral Ischemia. *PLoS One* **11**, e0158157 (2016).
51. The MICAD Research Team. 2'-[18F]Fluoroflumazenil. in *Molecular Imaging and Contrast Agent Database (MICAD)* (National Center for Biotechnology Information (US), Bethesda (MD), 2004).
52. Moresco, R. M. *et al.* Effects of fluvoxamine treatment on the in vivo binding of [F-18]FESP in drug naive depressed patients: a PET study. *Neuroimage* **12**, 452–465 (2000).
53. Barrio, J. R. *et al.* 3-(2'-[18F]fluoroethyl)spiperone: in vivo biochemical and kinetic characterization in rodents, nonhuman primates, and humans. *J Cereb Blood Flow Metab* **9**, 830–839 (1989).
54. Leung, K. 3-N-(2-[18F]Fluoroethyl)spiperone. in *Molecular Imaging and Contrast Agent Database (MICAD)* (National Center for Biotechnology Information (US), Bethesda (MD), 2004).
55. Brust, P. *et al.* In vivo Measurement of the Serotonin Transporter with (S)-([18F]fluoromethyl)-(+)-McN5652. *Neuropsychopharmacology* **28**, 2010–2019 (2003).
56. Hesse, S. *et al.* Imaging of the brain serotonin transporters (SERT) with 18F-labelled fluoromethyl-McN5652 and PET in humans. *European Journal of Nuclear Medicine and Molecular Imaging* **39**, 1001–1011 (2012).
57. Chakhoyan, A. *et al.* FMISO-PET-derived brain oxygen tension maps: application to glioblastoma and less aggressive gliomas. *Sci Rep* **7**, 10210 (2017).
58. Martínez, G. *et al.* 18F PET with flutemetamol for the early diagnosis of Alzheimer's disease dementia and other dementias in people with mild cognitive impairment (MCI). *Cochrane Database Syst Rev* **2017**, CD012884 (2017).
59. Nikolaus, S. *et al.* In vivo measurement of D2 receptor density and affinity for 18F-(3-N-methyl)benperidol in the rat striatum with a PET system for small laboratory animals. *J Nucl Med* **44**, 618–624 (2003).
60. Brašić, J. R. *et al.* Reduced Expression of Cerebral Metabotropic Glutamate Receptor Subtype 5 in Men with Fragile X Syndrome. *Brain Sci* **10**, 899 (2020).
61. Ravert, H. T., Scheffel, U., Mathews, W. B., Musachio, J. L. & Dannals, R. F. [11C]-GR89696, a potent kappa opiate receptor radioligand; *in vivo* binding of the R and S enantiomers. *Nuclear Medicine and Biology* **29**, 47–53 (2002).

62. Talbot, P. S. *et al.* 11C-GR103545, a radiotracer for imaging kappa-opioid receptors in vivo with PET: synthesis and evaluation in baboons. *J. Nucl. Med.* **46**, 484–494 (2005).
63. Handschuh, P. A. *et al.* Effect of MAOA DNA Methylation on Human in Vivo Protein Expression Measured by [11C]harmine Positron Emission Tomography. *Int J Neuropsychopharmacol* **26**, 116–124 (2023).
64. al-Tikriti, M. S. *et al.* Characterization of the dopamine transporter in nonhuman primate brain: homogenate binding, whole body imaging, and ex vivo autoradiography using [125I] and [123I]IPCIT. *Nucl Med Biol* **22**, 649–658 (1995).
65. Scanley, B. E. *et al.* Comparison of [123I]beta-CIT and [123I]IPCIT as single-photon emission tomography radiotracers for the dopamine transporter in nonhuman primates. *Eur J Nucl Med* **22**, 4–11 (1995).
66. Scanley, B. E. *et al.* [123I]IPCIT and [123I]beta-CIT as SPECT tracers for the dopamine transporter: a comparative analysis in nonhuman primates. *Nucl Med Biol* **27**, 13–21 (2000).
67. Froklage, F. E. *et al.* Quantification of 11C-Laniquidar Kinetics in the Brain. *J Nucl Med* **56**, 1730–1735 (2015).
68. Zoghbi, S. S. *et al.* 11C-loperamide and its N-desmethyl radiometabolite are avid substrates for brain permeability-glycoprotein efflux. *J Nucl Med* **49**, 649–656 (2008).
69. Parsey, R. V. *et al.* In vivo quantification of brain serotonin transporters in humans using [11C]McN 5652. *J Nucl Med* **41**, 1465–1477 (2000).
70. Smith, D. F. *et al.* Effects of pyrroloisoquinoline enantiomers ((+)- and (-)-McN-5652-Z) on behavioral and pharmacological serotonergic mechanisms in rats. *Eur J Pharmacol* **196**, 85–92 (1991).
71. Schreiber, R., Brocco, M. & Millan, M. J. Blockade of the discriminative stimulus effects of DOI by MDL 100,907 and the ‘atypical’ antipsychotics, clozapine and risperidone. *Eur J Pharmacol* **264**, 99–102 (1994).
72. Ullrich, T. & Rice, K. C. A practical synthesis of the serotonin 5-HT2A receptor antagonist MDL 100907, its enantiomer and their 3-phenolic derivatives as precursors for [11C]labeled PET ligands. *Bioorg Med Chem* **8**, 2427–2432 (2000).
73. Rami-Mark, C. *et al.* Radiosynthesis and first preclinical evaluation of the novel norepinephrine transporter pet-ligand [(11)C]ME@HAPTHI. *EJNMMI Res* **5**, 113 (2015).
74. Dumanic, M. *et al.* Evaluation of the cardiac neuronal function after acute myocardial infarction in rats using [11C]Me@HAPTHI, a novel [11C]-labeled PET tracer for the norepinephrine transporter. *Journal of Nuclear Medicine* **57**, 400–400 (2016).

75. Wu, X. *et al.* [11C]metomidate PET-CT versus adrenal vein sampling for diagnosing surgically curable primary aldosteronism: a prospective, within-patient trial. *Nat Med* **29**, 190–202 (2023).

76. Vraka, C. *et al.* Optimization of the Automated Synthesis of [11C]mHED-Administered and Apparent Molar Activities. *Pharmaceuticals (Basel)* **12**, (2019).

77. Beitzke, D. *et al.* Assessment of sympathetic reinnervation after cardiac transplantation using hybrid cardiac PET/MRI: A pilot study. *Journal of Magnetic Resonance Imaging* **50**, 1326–1335 (2019).

78. Frey, B. N., Rosa-Neto, P., Lubarsky, S. & Diksic, M. Correlation between serotonin synthesis and 5-HT1A receptor binding in the living human brain: a combined alpha-[11C]MT and [18F]MPPF positron emission tomography study. *Neuroimage* **42**, 850–857 (2008).

79. Kitamura, S. *et al.* Serotonergic Neurotransmission in Limbic Regions May Reflect Therapeutic Response of Depressive Patients: A PET Study With 11C-WAY-100635 and 18F-MPPF. *Int J Neuropsychopharmacol* **26**, 474–482 (2023).

80. Betthauser, T. J. *et al.* Human biodistribution and dosimetry of [18F]nifene, an α4β2* nicotinic acetylcholine receptor PET tracer. *Nuclear Medicine and Biology* **55**, 7–11 (2017).

81. Hillmer, A. T. *et al.* PET Imaging of 4 2* Nicotinic Acetylcholine Receptors: Quantitative Analysis of 18F-Nifene Kinetics in the Nonhuman Primate. *Journal of Nuclear Medicine* **53**, 1471–1480 (2012).

82. Weerts, E. M. *et al.* Differences in delta- and mu-opioid receptor blockade measured by positron emission tomography in naltrexone-treated recently abstinent alcohol-dependent subjects. *Neuropsychopharmacology* **33**, 653–665 (2008).

83. Abi-Dargham, A. *et al.* Measurement of Striatal and Extrastriatal Dopamine D1 Receptor Binding Potential With [11C]NNC 112 in Humans: Validation and Reproducibility: *Journal of Cerebral Blood Flow & Metabolism* 225–243 (2000) doi:10.1097/00004647-200002000-00003.

84. Halldin, C. *et al.* [(11)C]PE2I: a highly selective radioligand for PET examination of the dopamine transporter in monkey and human brain. *Eur J Nucl Med Mol Imaging* **30**, 1220–1230 (2003).

85. Ginovart, N. *et al.* Positron emission tomography quantification of [11C]-(+)-PHNO binding in the human brain. *J. Cereb. Blood Flow Metab.* **27**, 857–871 (2007).

86. Planas, A. M. *et al.* Rat brain acetylcholinesterase visualized with [11C]physostigmine. *Neuroimage* **1**, 173–180 (1994).

87. Shinotoh, H., Fukushi, K., Nagatsuka, S. & Irie, T. Acetylcholinesterase imaging: its use in therapy evaluation and drug design. *Curr Pharm Des* **10**, 1505–1517 (2004).

88. Pappata, S. *et al.* In vivo imaging of human cerebral acetylcholinesterase. *J Neurochem* **67**, 876–879 (1996).

89. Chauveau, F., Boutin, H., Van Camp, N., Dollé, F. & Tavitian, B. Nuclear imaging of neuroinflammation: a comprehensive review of [11C]PK11195 challengers. *Eur J Nucl Med Mol Imaging* **35**, 2304–2319 (2008).

90. Leung, K. 1-[11C]Methylpiperidin-4-yl propionate. in *Molecular Imaging and Contrast Agent Database (MICAD)* (National Center for Biotechnology Information (US), Bethesda (MD), 2004).

91. Kilbourn, M. R., Snyder, S. E., Sherman, P. S. & Kuhl, D. E. In vivo studies of acetylcholinesterase activity using a labeled substrate, N-[11C]methylpiperdin-4-yl propionate ([11C]PMP). *Synapse* **22**, 123–131 (1996).

92. Farde, L., Eriksson, L., Blomquist, G. & Halldin, C. Kinetic Analysis of Central [11C]Raclopride Binding to D2-Dopamine Receptors Studied by PET—A Comparisonto the Equilibrium Analysis. *Journal of Cerebral Blood Flow & Metabolism* **9**, 696–708 (1989).

93. Volkow, N. D. *et al.* Imaging endogenous dopamine competition with [11C]raclopride in the human brain. *Synapse* **16**, 255–262 (1994).

94. Patt, M. *et al.* Synthetic approaches and bio-distribution studies of [11C]methyl-phenidate. *J Pharm Pharm Sci* **10**, 312s–320s (2007).

95. Kenk, M. *et al.* PET measurements of cAMP-mediated phosphodiesterase-4 with (R)-[11C]rolipram. *Curr Radiopharm* **4**, 44–58 (2011).

96. Fujita, M. *et al.* Quantification of brain phosphodiesterase 4 in rat with (R)-[11C]Rolipram-PET. *NeuroImage* **26**, 1201–1210 (2005).

97. Fujita, M. *et al.* Downregulation of brain phosphodiesterase type IV measured with 11C-(R)-rolipram positron emission tomography in major depressive disorder. *Biol Psychiatry* **72**, 548–554 (2012).

98. Marner, L. *et al.* Kinetic Modeling of 11C-SB207145 Binding to 5-HT4 Receptors in the Human Brain In Vivo. *Journal of Nuclear Medicine* **50**, 900–908 (2009).

99. Marner, L. *et al.* [18F]FE-PE2I PET is a feasible alternative to [123I]FP-CIT SPECT for dopamine transporter imaging in clinically uncertain parkinsonism. *EJNMMI Res* **12**, 56 (2022).

100. Farde, L., Halldin, C., Stone-Elander, S. & Sedvall, G. PET analysis of human dopamine receptor subtypes using 11C-SCH 23390 and 11C-raclopride. *Psychopharmacology* **92**, 278–284 (1987).

101. Frey, K. A. *et al.* In vivo muscarinic cholinergic receptor imaging in human brain with [11C]scopolamine and positron emission tomography. *J Cereb Blood Flow Metab* **12**, 147–154 (1992).

102. Blin, J. *et al.* Loss of brain 5-HT2 receptors in Alzheimer's disease. In vivo assessment with positron emission tomography and [18F]setoperone. *Brain* **116 ( Pt 3)**, 497–510 (1993).

103. Beversdorf, D. Q. *et al.* 5-HT2 receptor distribution shown by [18F] setoperone PET in high-functioning autistic adults. *J Neuropsychiatry Clin Neurosci* **24**, 191–197 (2012).

104. Philippe, C. *et al.* Preclinical in vitro &in vivo evaluation of [(11)C]SNAP-7941 - the first PET tracer for the melanin concentrating hormone receptor 1. *Nucl. Med. Biol.* **40**, 919–925 (2013).

105. Vraka, C. *et al.* A new Method Measuring the Interaction of Radiotracers with the human P-glycoprotein (P-gp) Transporter. *Nuclear Medicine and Biology* 29–36 (2018) doi:10.1016/j.nucmedbio.2018.02.002.

106. Naganawa, M. *et al.* Test-retest variability of adenosine A2A binding in the human brain with (11)C-TMSX and PET. *EJNMMI Res* **4**, 76 (2014).

107. Bauer, M. *et al.* Pgp-mediated interaction between (R)-[11C]verapamil and tariquidar at the human blood-brain barrier: a comparison with rat data. *Clin. Pharmacol. Ther.* **91**, 227–233 (2012).

108. Farde, L., Ito, H., Swahn, C. G., Pike, V. W. & Halldin, C. Quantitative analyses of carbonyl-carbon-11-WAY-100635 binding to central 5-hydroxytryptamine-1A receptors in man. *J. Nucl. Med.* **39**, 1965–1971 (1998).
